# Antitumor immunity augments the therapeutic effects of p53 activation on acute myeloid leukemia

**DOI:** 10.1038/s41467-019-12555-1

**Published:** 2019-10-25

**Authors:** Yasutaka Hayashi, Susumu Goyama, XiaoXiao Liu, Moe Tamura, Shuhei Asada, Yosuke Tanaka, Tomofusa Fukuyama, Mark Wunderlich, Eric O’Brien, Benjamin Mizukawa, Satoshi Yamazaki, Akiko Matsumoto, Satoshi Yamasaki, Tatsuhiro Shibata, Koichi Matsuda, Goro Sashida, Hitoshi Takizawa, Toshio Kitamura

**Affiliations:** 1 Division of Cellular Therapy, The Institute of Medical Science, The University of Tokyo, Tokyo, Japan; 2 Cancer & Blood Diseases Institute, Cincinnati Children’s Hospital Medical Center, University of Cincinnati College of Medicine, Cincinnati, OH USA; 3 Division of Stem Cell Therapy, The Institute of Medical Science, The University of Tokyo, Tokyo, Japan; 4 Laboratory of Molecular Medicine, The Institute of Medical Science, The University of Tokyo, Tokyo, Japan; 5 Laboratory of Clinical Genome Sequencing, Department of Computational biology and medical Sciences, Graduate school of Frontier Sciences, The University of Tokyo, Tokyo, Japan; 6 Laboratory of Transcriptional Regulation in Leukemogenesis, International Research Center for Medical Sciences, Kumamoto University, Kumamoto, Japan; 7 Laboratory of Stem Cell Stress, International Research Center for Medical Sciences, Kumamoto University, Kumamoto, Japan

**Keywords:** Cancer immunotherapy, Acute myeloid leukaemia

## Abstract

The negative regulator of p53, MDM2, is frequently overexpressed in acute myeloid leukemia (AML) that retains wild-type *TP53* alleles. Targeting of p53-MDM2 interaction to reactivate p53 function is therefore an attractive therapeutic approach for AML. Here we show that an orally active inhibitor of p53-MDM2 interaction, DS-5272, causes dramatic tumor regressions of MLL-AF9-driven AML in vivo with a tolerable toxicity. However, the antileukemia effect of DS-5272 is markedly attenuated in immunodeficient mice, indicating the critical impact of systemic immune responses that drive p53-mediated leukemia suppression. In relation to this, DS-5272 triggers immune-inflammatory responses in MLL-AF9 cells including upregulation of Hif1α and PD-L1, and inhibition of the Hif1α-PD-L1 axis sensitizes AML cells to p53 activation. We also found that NK cells are important mediators of antileukemia immunity. Our study showed the potent activity of a p53-activating drug against AML, which is further augmented by antitumor immunity.

## Abstract (web summary)

MDM2 is frequently overexpressed in acute myeloid leukaemia leading to p53 inactivation. Here, the authors are demonstrating that an inhibitor of p53-MDM2 interaction, DS-5272, induce in vivo tumour regression through immune response regulation.

## Introduction

The tumor suppressor protein p53, encoded by *TP53* gene in humans, plays an important role in preventing cancer development^1,2^. More than half of cancers have mutations in the *TP53* gene. In addition, activity of wild-type p53 is often suppressed in the remaining cancers due to overexpression of p53-regulatory proteins. The principal cellular antagonist of p53 is an E3 ubiquitin ligase MDM2^3,4^. MDM2 binds to p53 and induces its proteasomal degradation. Therefore, p53 activation using small-molecule inhibitors of the p53-MDM2 interaction has been regarded as an attractive strategy to treat cancers harboring wild-type p53^5,6^. DS-5272 is one of the p53-MDM2 interaction inhibitors that shows robust antitumor activity in vivo^7^.

Acute myeloid leukemia (AML) is a blood cancer with uncontrolled overproduction of myeloid cells^8^. The reported frequency of *TP53* mutation is relatively low (5–10%), but dysfunction of p53 pathway is highly prevalent in AML^9^. Elevated MDM2 expression occurs in over a third of patients with AML, who have low levels of p53 protein and suffer from poor clinical outcomes similar to patients with *TP53* mutations. Previous studies have also shown that p53 is functionally inactivated^10–13^, but is rarely mutated in AML with *MLL* rearrangements^14^. These findings suggest that AMLs with MDM2 overexpression and/or *MLL* rearrangements could be highly susceptible to p53-activating drugs.

The host immune system serves as a barrier to inhibit tumor formation and progression. Treatments targeting immune checkpoint molecules, including PD-1 and its ligand PD-L1, have been approved for treating human cancers with durable clinical benefit^15^. It is widely accepted that checkpoint blockade unleashes cytotoxic T-lymphocytes (CTLs) attack tumor cells. In addition, recent reports have shown the contribution of NK cells to mediate the effect of PD-1/PD-L1 blockade immunotherapy^16^. Several upstream regulators of PD-L1, such as Myc^17^, CDKs^18–20^, and Hif1α^21^, have been identified as potential targets to enhance the effect of immunotherapy. Studies have also shown that p53 in tumor cells communicates with CTLs to promote CTL-induced tumor cell death^22^. However, the role of p53 in the regulation of NK cell function remains unknown.

In this study, we show the potent antileukemia effect of DS-5272 using a mouse AML model driven by MLL-AF9 and patient-derived xenograft (PDX) models of human AML^23^. MLL-AF9 is one of the most prevalent forms of MLL-fusion oncogene, and has the ability to transform both human and mouse hematopoietic progenitor cells into AML cells^24–26^. Importantly, the antileukemia effect of DS-5272 is attenuated in immunodeficient mice and immunocompetent mice with NK cell depletion. Furthermore, inhibition of Hif1α-PD-L1 axis enhances the therapeutic efficacy of DS-5272. These data suggest that pharmacological activation of p53 exerts the potent antileukemia effect with the assistance of antitumor immunity, including NK cell-mediated cytotoxicity against AML.

## Results

### p53 activation inhibits the growth of mouse MLL-AF9 cells

**Fig. 1:**
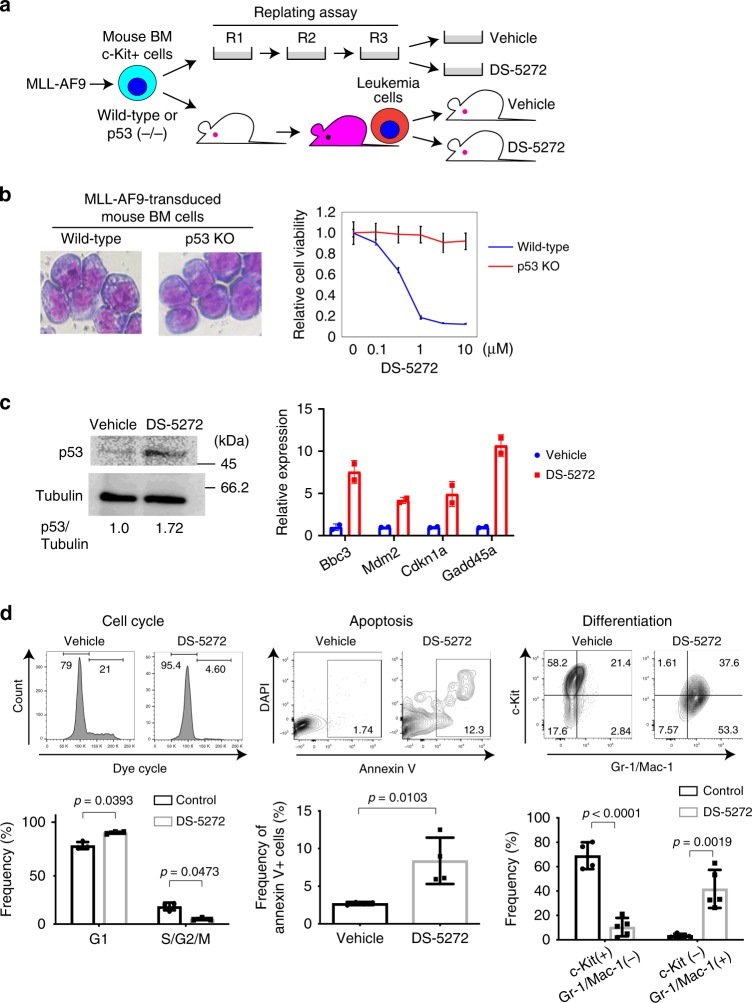
DS-5272 activates p53 and inhibits the growth of MLL-AF9 cells both in vitro and in vivo. **a** Experimental scheme used in **b**–**d**. Bone marrow c-Kit + cells derived from wild-type or p53-deficient mice were retrovirally transduced with MLL-AF9. The cells were serially replated in vitro or directly transplanted into recipient mice to produce MLL-AF9 leukemia cells. **b** (left) Wright-Giemsa staining of wild-type and p53-deficient leukemia cells induced by MLL-AF9. Original magnification, ×40. (right) Viability of cells treated with DS-5272 at indicated concentrations was measured by WST-8 assay. Data are normalized to vehicle control (0 μM group), and are shown as mean ± s.d. **c** GFP+ MLL-AF9 leukemia cells were collected from bone marrows of vehicle- or DS-5272-treated mice. Expression levels of p53 protein (left) and p53-target genes (right) were assessed by western blotting or qPCR, respectively. Band intensities of p53 and Tubulin were quantified with Multi Gauge (Science Lab), and the intensities of p53 relative to GAPDH are shown. **d** Cell-cycle status, apoptosis, and differentiation were assessed in GFP + MLL-AF9 leukemia cells derived from vehicle- or DS-5272-treated mice 24 h after treatment. Representative FACS plots (upper panels) and their quantification for three to four independent mice (lower panels). (Cell cycle_Vehicle: *n* = 3, DS-5272: *n* = 3, Apoptosis_Vehicle: *n* = 4, DS-5272: *n* = 4, Differentiation_Vehicle: *n* = 4, DS-5272: *n* = 5). Experiments were independently repeated at least three times. Student *t*-test. The numbers shown in upper panels indicate the percentage of cells within each gate. Data are shown as mean ± s.d. in lower panels

We first assessed the effect of DS-5272 using a mouse AML model driven by MLL-AF9. Bone marrow (BM) progenitors derived from wild-type or p53-deficient mice were transduced with MLL-AF9 (coexpressing GFP), and were serially replated in semisolid medium or directly transplanted into recipient mice (Fig. 1a). DS-5272 inhibited in vitro growth of p53-intact MLL-AF9 leukemia cells with the IC50 value in the nanomolar range. In contrast, p53-deficient MLL-AF9 cells were resistant to DS-5272 even at higher concentrations, confirming that p53 is required for the growth-inhibitory effect of DS-5272 (Fig. 1b, Supplementary Fig. 1). We then treated recipient mice that received MLL-AF9 leukemia cells with vehicle or DS-5272 10 days after transplantation. Single dose administration of DS-5272 induced upregulation of p53 protein and p53-target genes in MLL-AF9 cells in vivo (Fig. 1c). Furthermore, the DS-5272-mediated p53 activation induced cell cycle arrest, apoptosis, and differentiation of MLL-AF9 cells (Fig. 1d). DS-5272 treatment did not increase levels of reactive oxygen species (ROS) in MLL-AF9 cells, indicating that these antileukemia effects are independent of ROS overproduction (Supplementary Fig. 2).

**Fig. 2:**
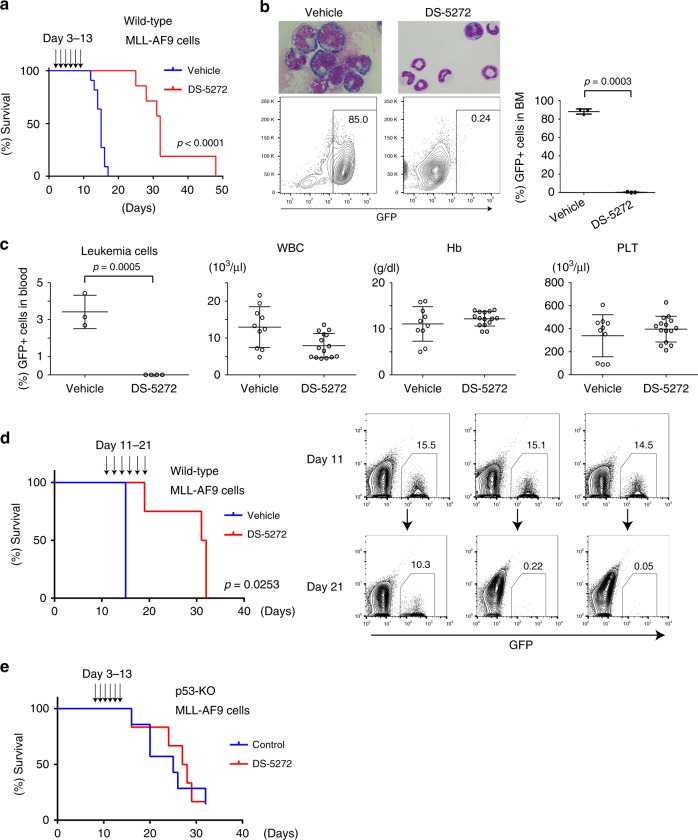
DS-5272 inhibits the development of MLL-AF9-driven leukemia in vivo. **a** Kaplan–Meier survival curves of MLL-AF9 leukemia mice treated with vehicle or DS-5272 from day 3 to day 13. (*n* = 12 per group). Statistical significance was evaluated by the log rank test. **b** Representative images and FACS analyses of BM cells derived from vehicle or DS-5272-treated MLL-AF9 leukemia mice (left), and the summarized data for three biologically independent experiments (right). Student *t*-test. **c** Frequency of GFP + leukemia cells in peripheral blood (Control: *n* = 3, DS-5272: *n* = 4), white blood cell counts (WBC), hemoglobin levels (Hb) and platelet counts (PLT) in leukemic mice treated with vehicle or DS-5272 at day 10. (Vehicle: *n* = 10, DS-5272: *n* = 15, data are shown as mean ± s.d.). **d** (left) Kaplan–Meier survival curves of MLL-AF9 leukemia mice treated with vehicle or DS-5272 from day 11 to day 21. (Control: *n* = 2, DS-5272: *n* = 4). Statistical significance was evaluated by the log rank test. (right) FACS plots of peripheral blood cells collected from MLL-AF9 leukemia mice at day 11 and day 21, showing reduction of GFP + leukemia cells at day 21 in the DS-5272-treated mice. **e** Kaplan–Meier survival curves of mice injected with p53-deficient MLL-AF9 cells, that were treated with vehicle or DS-5272 from day 3 to day 13. (Control: *n* = 7, DS-5272: *n* = 6)

Next, we treated leukemia mice with vehicle or DS-5272 on an every 2-day schedule from day 3 to day 13. Mice treated with DS-5272 showed significantly prolonged survival, which continued well beyond discontinuation of therapy (Fig. 2a). DS-5272 treatment also led to dramatic decrease in the leukemic involvement of their BM and peripheral blood, whereas it showed only a little adverse effect on normal hematopoiesis (Fig. 2b, c, Supplementary Fig. 3). Delayed initiation of DS-5272 (from day 11 to day 21) still prolonged the survival of MLL-AF9 leukemia mice, with reduction of GFP+ leukemia cells in peripheral blood and improvement of hind limb paralysis of leukemic mice (Fig. 2d and Supplementary Movie 1-4). Thus, DS-5272 showed robust and strong antileukemia effect against p53-intact MLL-AF9 leukemia. As expected, DS-5272 did not inhibit the development of p53-deficient MLL-AF9 leukemia in vivo (Fig. 2e).

### p53 provokes inflammatory responses in leukemia cells

**Fig. 3:**
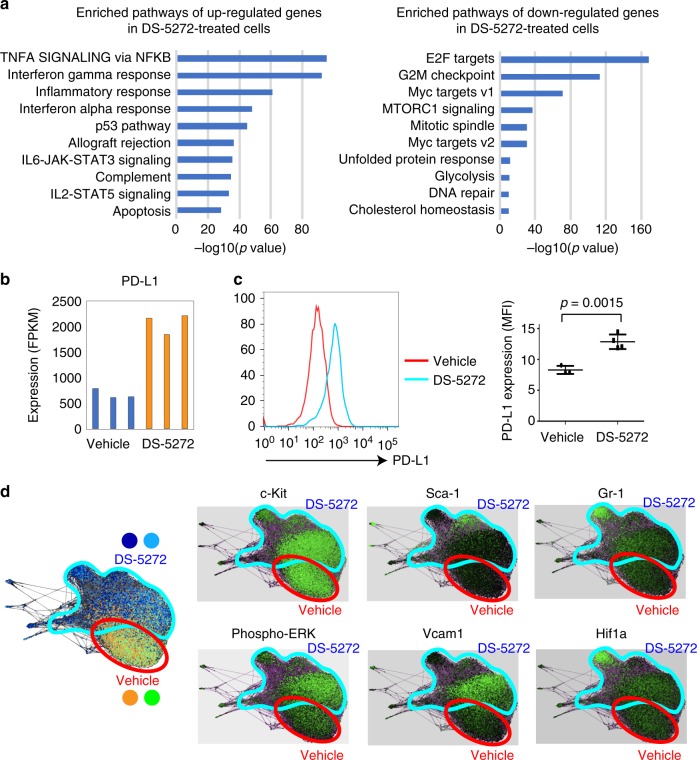
DS-5272 treatment triggers an immune-inflammatory response in MLL-AF9 cells. **a** GSEA for up- and down-regulated genes in DS-5272-treated MLL-AF9 cells using the Hallmark collections of the GSEA MSigDB (http://software.broadinstitute.org/gsea/msigdb). The *x* axis shows the *P* value (−log_10_). **b** mRNA expression of PD-L1 in vehicle- or DS-5272-treated MLL-AF9 cells. **c** Cell surface expression of PD-L1 in vehicle- or DS-5272-treated MLL-AF9 cells. Representative FACS plots (left) and quantification of MFI of PD-L1 in MLL-AF9 cells (right). Data are shown as mean ± s.d. of four biologically independent experiments. Student t-test. **d** 25-dimensional analysis using SPRING (https://kleintools.hms.harvard.edu/tools/spring.html) of bone marrow cells from MLL-AF9 leukemia mice treated with vehicle and DS-5272 for 24 h (*n* = 2/condition). Differences in the location of cells within the SPRING map resulted from changes in protein expression. The regions enclosed by the red and blue curves represent the cells treated with vehicle or DS-5272, respectively. At right, surface (c-Kit, Gr-1, Vcam1) and intracellular signaling pathway (phospho-ERK) markers in vehicle- and DS-5272-treated MLL-AF9 leukemia cells are shown

To assess the molecular changes induced by p53 activation in MLL-AF9 leukemia, we first examined expression profiles of vehicle- or DS-5272-treated MLL-AF9 cells. Consistent with earlier results, RNA-Seq and subsequent pathway analyses using GSEA^27,28^ revealed upregulation of p53- and apoptosis-related genes as well as downregulation of cell cycle regulatory genes upon DS-5272 treatment (Fig. 3a). DS-5272 treatment also induced upregulation of inflammation- and interferon-associated genes in MLL-AF9 cells, including PD-L1 that plays a critical role in suppressing T and NK cell activation^15,16^ (Fig. 3a–c, Supplementary Fig. 4). These expression changes suggest that p53 activation triggers an immune-inflammatory response that leads to leukemia regression, and the upregulated PD-L1 in surviving leukemia cells promotes their immune escape.

We then assessed changes of surface marker expression and signal transduction induced by DS-5272 in MLL-AF9 cells using the single-cell mass cytometry. Analyses of the mass cytometry data using SPRING^29^ and SPADE^30^ revealed distinct molecular signatures between vehicle- and DS-5272-treated cells, including upregulation of Gr-1 and Sca-1 in subsets of DS-5272-treated leukemia cells. DS-5272 also induced activation of ERK pathway and upregulation of an adhesion molecule VCAM-1^31^ in c-Kit + population (Fig. 3d). Expression of other cell surface molecules that were shown to regulate hematopoietic and leukemia stem cells, EPCR, CXCR4 and CD49d, were not upregulated by DS-5272 (Supplementary Fig. 5A). Furthermore, we found strong activation of Hif1α, Stat1 and Stat4 pathways in Gr-1+ population upon DS-5272 treatment, indicating the enhanced inflammatory signaling in the relatively differentiated leukemia cells in agreement with the RNA-seq data above (Fig. 3d, Supplementary Fig. 5B, Supplementary Fig. 6). The Gr-1+ MLL-AF9 cells retained colonogenic activity (Supplementary Fig. 7), suggesting that they are not terminally differentiated cells. These Gr-1+ leukemia cells may play important roles to suppress T and/or NK cell functions as myeloid-derived-suppressor cells (MDSCs)^32^ do in solid tumors.

**Fig. 4:**
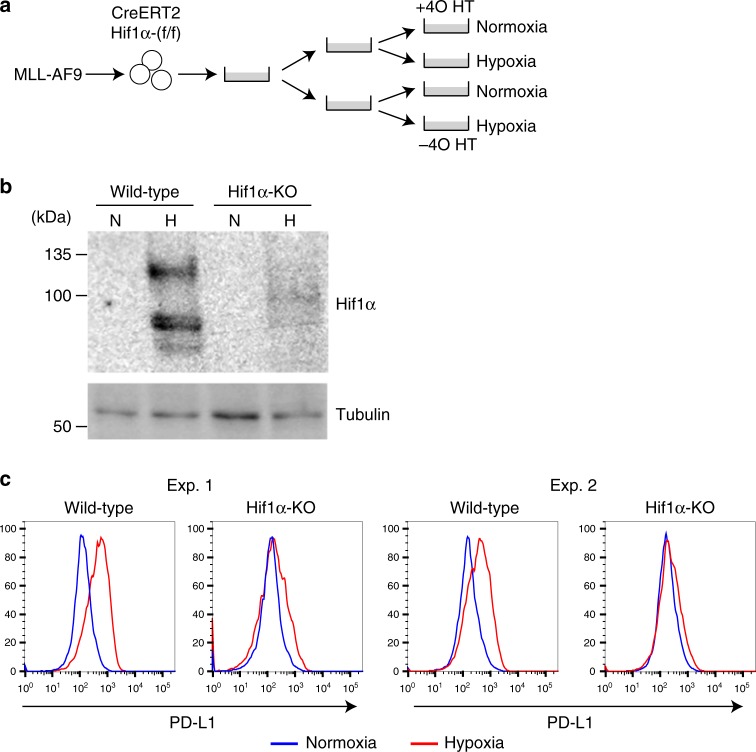
PD-L1 is a downstream target of Hif1α in MLL-AF9 cells. **a** Experimental scheme used in **b**, **c**. Bone marrow c-Kit + cells derived from Hif1α^flox/flox^; Rosa26-Cre-ERT2 mice were transduced with MLL-AF9, and were treated with EtOH or 1 μM 4OHT in semisolid media. The Hif1α-intact and Hif1α-deleted cells were then cultured under either hypoxia or normoxia condition for 24 h. **b**, **c** Cells described in **a** were subjected to immunoblotting with Hif1α and tubulin antibodies (**b**), and FACS analyses to evaluate PD-L1 expression (**c**)

Because PD-L1 was shown to be a transcriptional target of Hif1α in MDSCs^21^, we examined if PD-L1 expression is dependent on Hif1α in MLL-AF9 cells. We transduced MLL-AF9 into bone marrow c-Kit + cells derived from Hif1α^flox/flox^; Rosa26-Cre-ERT2 mice to establish an inducible gene knockout system for *Hif1*α (Fig. 4a). Exposure of control MLL-AF9 cells to hypoxia resulted in upregulation of both Hif1α and PD-L1. This hypoxia-induced PD-L1 upregulation was not observed in Hif1α-depleted cells (Fig. 4b, c), indicating that PD-L1 is a downstream target of Hif1α in MLL-AF9 cells. Taken together, these results suggest that pharmacological activation of p53 provokes inflammatory and immune responses in MLL-AF9 cells, including activation of Hif1a-PD-L1 pathway.

### Therapy-resistant cells are enriched in BM endosteal region

**Fig. 5:**
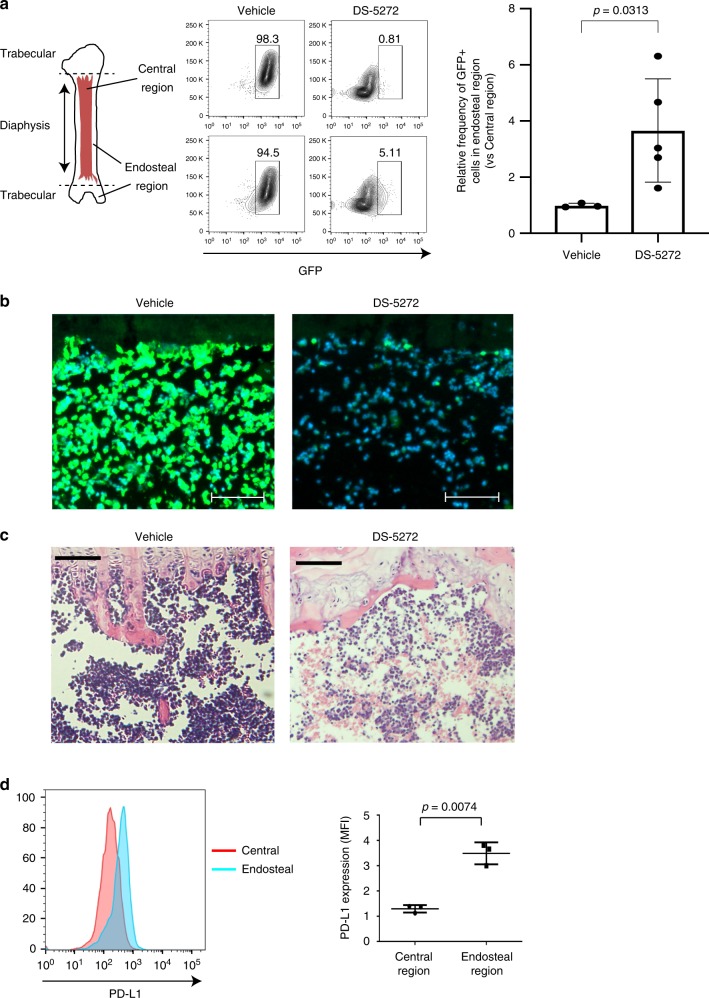
Therapy-resistant leukemia cells are enriched in endosteal BM region. **a** Mice transplanted with MLL-AF9 leukemia cells were treated with vehicle or DS-5272 for 2 weeks. Cells in central and endosteal BM regions were isolated from these mice, and were analyzed by FACS. Shown are frequencies of GFP + leukemia cells in central and endosteal BM regions. Representative FACS plots (left) and their quantification for three (Vehicle) or five (DS-5272) independent mice (right). The numbers shown indicate the percentage of cells within each gate (left), and data are shown as mean ± s.d. (right). Student *t*-test. **b** Bone marrow progenitors derived from C57BL/6-Tg (CAG-EGFP) mice were transduced with MLL-AF9, and were transplanted into recipient mice. These mice were treated with vehicle or DS-5272, and distribution of GFP + leukemia cells in femur was analyzed with microscopy 24 h after treatment. GFP + leukemia cells were markedly reduced and were enriched in endosteal regions in DS-5272-treated mice. 10x magnification, scale bar, 100 μm. **c** Mice transplanted with MLL-AF9 leukemia cells were treated with vehicle control or DS-5272. The tibiae were resected 24 h after treatment and histologically processed to obtain H&E stained sections. ×10 magnification, scale bar, 100 μm. **d** Cell surface expression of PD-L1 in MLL-AF9 cells collected from central or endosteal BM regions. Representative FACS plots (left) and quantification of MFI of PD-L1 in MLL-AF9 cells (right). Data are shown as mean ± s.d. of three biologically independent experiments. Student *t*-test

Despite the remarkable therapeutic effect of DS-5272, all leukemia mice eventually relapsed ~2 weeks following discontinuation of treatment. MLL-AF9 cells derived from relapsed mice after the initial therapy did not have mutations in *Trp53* gene, but were no longer sensitive to DS-5272 in vivo (Supplementary Fig. 8). To examine spatial distribution of therapy-resistant leukemia cells in BM, we next assessed the frequency of GFP+ leukemia cells in trabecular-rich endosteal BM areas and central BM areas^33^ with or without DS-5272 treatment. Most GFP+ leukemia cells were detected in endosteal BM regions where osteoblasts reside in DS-5272-treated mice. In contrast, both endosteal and central BM regions were filled with GFP+ leukemia cells in control mice (Fig. 5a). The enrichment of residual AML cells in endosteal BM region after DS-5272 treatment was also confirmed by microscopic observations (Fig. 5b, c). Thus, leukemia cells within the osteoblast-rich area appear to be protected from p53-induced cell death. Interestingly, leukemia cells reside in BM endosteal regions also expressed higher level of PD-L1 compared with those in BM central regions (Fig. 5d), which probably accounts for their low sensitivity to DS-5272. The high expression of PD-L1 in endosteal MLL-AF9 cells is likely associated with the increased inflammatory signaling in them, as was shown in a recent report^34^.

### Reduced antitumor effect of DS-5272 in immunodeficient mice

**Fig. 6:**
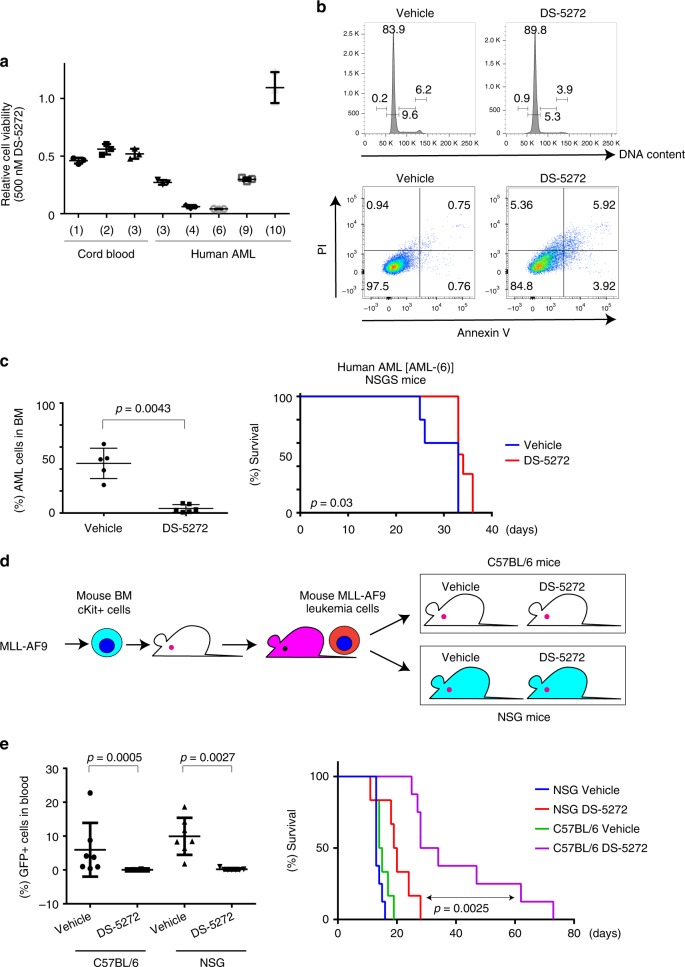
Limited antileukemia effects of DS-5272 in immunodeficient mice. **a** Viability of normal cord blood (CB) CD34 + cells and PDX-derived human AML cells treated with 500 nM DS-5272 was measured by WST-1 assay. Results are normalized to the viability of DMSO-treated cells, set at 1 (*n* = 3). Data are shown as the mean ± s.d. of triplicate wells. See also Supplementary Table 1 and Supplementary Fig. 11. **b** Cell-cycle status and apoptosis were assessed after 3 days culture of human AML-(6) cells with vehicle or DS-5272 (250 nM). (upper panels) Shown are cell cycle profiles assessed by FACS with PI staining of DNA. The numbers indicate the percentages of cells in the sub-G1, G1, S, and G2/M phases. (lower panels) Shown are FACS profiles of Annexin V and PI staining. The numbers indicate the percentages of Annexin V+ cells. See also Supplementary Fig. 9 and Supplementary Fig. 10. **c** (left) Frequency of human CD45+ AML cells in bone marrow of NSGS mice treated with vehicle or DS-5272 at day 20. Data are shown as mean ± s.d. of five or six biologically independent samples. (right) Kaplan–Meier survival curves of NSGS mice injected with AML-(6), that were treated with vehicle or DS-5272. (Vehicle: *n* = 5, DS-5272: *n* = 6) Statistical significance was evaluated by the log rank test. **d** Experimental scheme used in **e**. MLL-AF9-transduced mouse bone marrow cells were transplanted into C57BL/6 mice or NSG mice, and these mice were treated with either vehicle or DS-5272. **e** (left) Frequency of GFP + leukemia cells in peripheral blood of MLL-AF9 leukemia mice (C57BL/6 or NSG mice) treated with vehicle or DS-5272 at day 10. (NSG_Vehicle: *n* = 7, NSG_DS-5272: *n* = 8, C57BL/6_Vehicle: *n* = 7, C57BL/6_DS-5272: *n* = 8). Data are shown as mean ± s.d. of seven or eight independent samples. Student *t*-test. (right) Kaplan–Meier survival curves of C57BL/6 or NSG mice injected with MLL-AF9 cells, that were treated with vehicle or DS-5272. (*n* = 12 per group). Statistical significance was evaluated by the log rank test

We next assessed the therapeutic effect of DS-5272 using patient-derived xenograft (PDX) models for human AML^23^. We transplanted primary AML samples into NOD.Cg-*Prkdc*^*scid*^ *Il2rg*^*tm1Wjl*^Tg (CMV-IL3,CSF2,KITLG)1Eav/MloySzJ (NSGS) mice^35^ and established eleven PDXs for human AML with robust repopulating ability. We cultured these PDX-derived AML cells with DS-5272, and identified several AMLs that were more sensitive to DS-5272 than normal CD34 + cord blood cells (Fig. 6a). DS-5272 treatment induced apoptosis and/or cell cycle arrest in these human AML cells (Fig. 6b and Supplementary Figs. 9, 10). Consistent with previous reports^9,36,37^, approximately half of AML samples were sensitive, whereas cells with *TP53* mutations and several cell lines with wild-type *TP53* were resistant to DS-5272 (Supplementary Fig. 11A, Supplementary Table 1). MDM2 mRNA levels did not predict sensitivity of each PDX-AML to DS-5272 (Supplementary Fig. 11B), which is also consistent with previous reports^36,37^.

We then transplanted a DS-5272-sensitive PDX [AML-(6)] to NSGS mice and treated the mice with DS-5272. Similar to the earlier results obtained by the mouse AML model, DS-5272 treatment efficiently inhibited the engraftment of human AML cells in bone marrow at day 20. However, survival of these mice was only modestly improved (Fig. 6c). We speculated that the lack of an intact immune system could explain the limited survival benefit of NSGS mice treated with DS-5272 in this PDX model. To test this possibility, we compared the effect of DS-5272 on mouse MLL-AF9 cells in immunocompetent C57BL/6 mice and immunodeficient NSG mice (Fig. 6d). Although DS-5272 treatment initially inhibited leukemic progression of mouse MLL-AF9 cells in both mouse strains, it showed only modest effect to prolong the survival of NSG mice (Fig. 6e). These results suggest that the systemic immune response triggered by DS-5272 plays a crucial role in suppressing the regrowth of residual AML cells after the therapy, which is necessary for prolonged survival of leukemic mice treated with DS-5272.

### NK cells enhance the antileukemia effect of p53 activation

**Fig. 7:**
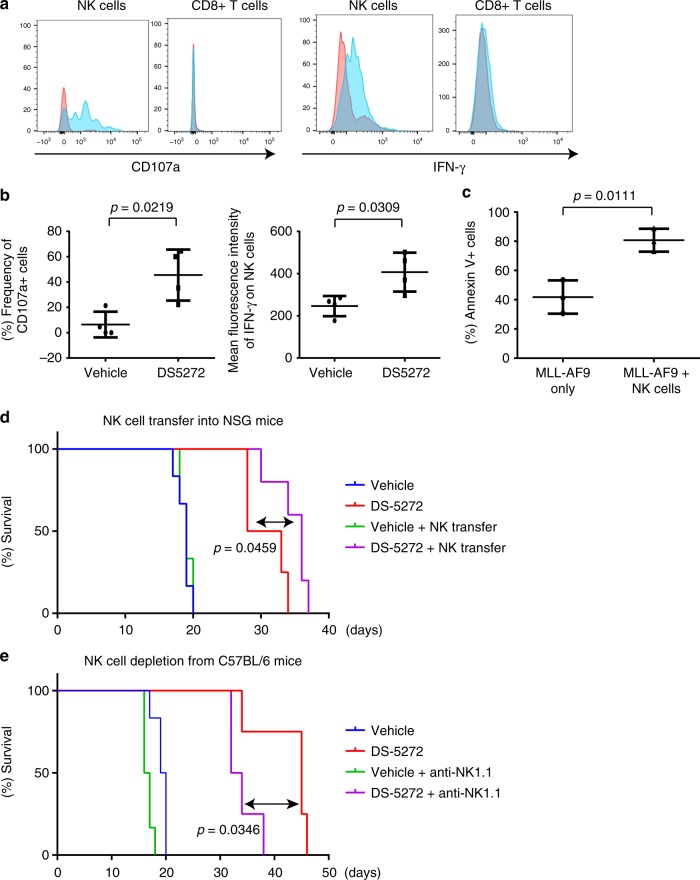
NK cells contribute to the antileukemia effect of DS-5272. **a** Representative FACS plots showing cell surface expression of Cd107a (left) and intracellular expression of IFN-γ (right) in NK cells and Cd8 + T cells in bone marrow. **b** Quantification of frequency of Cd107a+ cells (left) and mean fluorescence intensity (MFI) of IFN-γ (right) in NK cells. Data are shown as mean ± s.d. of four biologically independent samples. Student *t*-test. **c** MLL-AF9 leukemia cells were co-cultured with or without NK cells. Frequency of Annexin-V + cells in MLL-AF9 leukemia cells was assessed after 48 h of co-culture. Data are shown as mean ± s.d. of three biologically independent samples. Student t-test. **d** Kaplan–Meier survival curves of NSG mice injected with MLL-AF9 cells or MLL-AF9 cells + NK cells, that were treated with vehicle control or DS-5272. (Vehicle: n = 6, DS-5272: *n* = 4, Vehicle + NK: *n* = 6, DS-5272 + NK: *n* = 5). Statistical significance was evaluated by the log rank test. **e** Kaplan–Meier survival curves of C57BL/6J mice injected with MLL-AF9 cells, that were treated with vehicle control, anti-NK1.1, DS-5272 or DS-5272 + anti-NK1.1. (Vehicle: *n* = 6, DS-5272: *n* = 6, Vehicle + anti-NK1.1: *n* = 4, DS-5272 + anti-NK1.1: *n* = 4) Statistical significance was evaluated by the log rank test

To determine the major immune cell type that enhances the effect of p53 activation in vivo, we first examined the influence of DS-5272 treatment on activity of CD8 + T cells and NK cells in leukemia-bearing C57BL/6 mice. Interestingly, DS-5272 induced upregulation of active markers (CD107a and IFN-γ) in NK cells but not in CD8+ T cells (Fig. 7a, b). Furthermore, coculture of NK cells with mouse MLL-AF9 leukemia cells resulted in massive apoptosis of leukemia cells (Fig. 7c). These data suggest possible contribution of NK cells to DS-5272-mediaged leukemia suppression. To test this possibility, we next assessed the effect of NK cell transfer into immunodeficient NSG mice and that of NK cell depletion from immunocompetent C57BL/6 mice on the efficacy of DS-5272 using the MLL-AF9 leukemia model. NK cell transfer to NSG mice increased, whereas depletion of NK cells with anti-NK1.1 antibody from C57BL/6 mice reduced the therapeutic effect of DS-5272 against MLL-AF9 leukemia (Fig. 7d, e). These data suggest that NK cells are important mediators of antileukemia immunity during the treatment with DS-5272.

### Hif1α-PD-L1 axis limits the antileukemia effect of p53

**Fig. 8:**
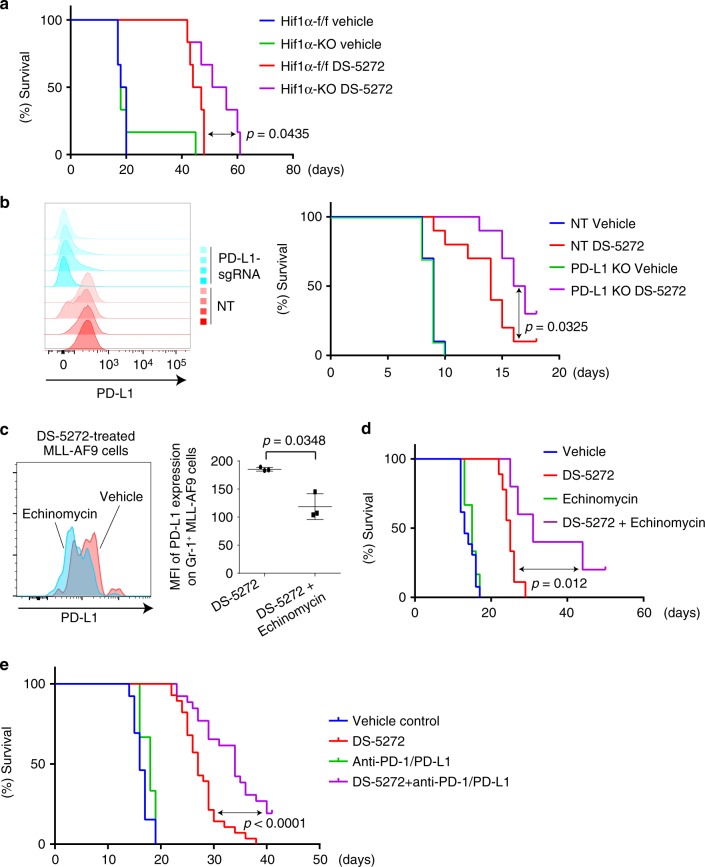
Inhibition of Hif1α-PD-L1 axis enhances the antileukemia effect of DS-5272. **a** MLL-AF9;CreERT2-Hif1α-f/f leukemia cells were established as described in Fig. 4a. The leukemia cells were pretreated with EtOH or 1 μM 4OHT for 24 h and were transplanted into recipient mice. Kaplan–Meier survival curves of these leukemic mice treated with vehicle or DS-5272. (*n* = 6 per group). Statistical significance was evaluated by the log rank test. **b** (left) Cell surface expression of PD-L1 in MLL-AF9 cells transduced with non-targeting (NT) control sgRNA or PD-L1-targeting sgRNA, showing efficient knockout (KO) of PD-L1. (right) Kaplan–Meier survival curves of mice injected with control (NT) or PD-L1-depleted (PD-L1 KO) MLL-AF9 cells, that were treated with vehicle or DS-5272 (*n* = 10 per group). Statistical significance was evaluated by the log rank test. **c** Representative FACS dot plots showing cell surface expression of PD-L1 (left), and quantitation of mean fluorescence intensity (MFI) of PD-L1 (right) in Gr-1+ MLL-AF9 cells treated with DS-5272 or DS-5272 + Echinomycin. Data are shown as mean ± s.d. of three biologically independent samples. Student *t*-test. **d** Kaplan–Meier survival curves of leukemic mice treated with vehicle, DS-5272, Echinomycin, or DS-5272 + Echinomycin (Vehicle: *n* = 13, DS-5272: *n* = 9, Echinomycin: *n* = 6, DS-5272 + Echinomycin: *n* = 5). Statistical significance was evaluated by the log rank test. **e** Kaplan–Meier survival curves of leukemic mice treated with vehicle, anti-PD-1/PD-L1, DS-5272, or DS-5272 + anti-PD-1/PD-L1. Vehicle: *n* = 16, DS-5272: *n* = 31, anti-PD-1/PD-L1: *n* = 6, DS-5272 + anti-PD-1/PD-L1: n = 29. Statistical significance was evaluated by the log rank test

Based on data described above, we hypothesized that AML cells expressing high levels of Hif1α and PD-L1 could evade immune attack and are therefore relatively resistant to DS-5272 treatment in vivo. To test this hypothesis, we genetically depleted Hif1α or PD-L1 in MLL-AF9 cells using Cre/loxP or CRISPR/Cas9 system, respectively. Neither Hif1α nor PD-L1 depletion inhibited the in vitro growth and in vivo development of MLL-AF9 leukemia, indicating that both genes are dispensable for leukemia progression in mice with no treatment. In contrast, MLL-AF9 cells deficient for Hif1α or PD-L1 became more sensitive to DS-5272 treatment than their counterparts (Fig. 8a, b, Supplementary Fig. 12), suggesting the important role of these genes to limit the effect of p53-activating therapy in vivo. We then assessed the combined effect of DS-5272 and the Hif1 inhibitor echinomycin^38^ on MLL-AF9-induced leukemogenesis. As expected, echinomycin alone did not inhibit leukemic progression of MLL-AF9 cells. However, echinomycin inhibited PD-L1 upregulation induced by DS-5272 in Gr-1+ MLL-AF9 cells (Fig. 8c), and combined treatment of DS-5272 and echinomycin significantly prolonged the survival of leukemia mice than treatment with either DS-5272 or echinomycin alone (Fig. 8d). Finally, we assessed the effect of combination therapy with DS-5272 and anti-PD-L1/PD-1 antibodies on survival time in mice transplanted with MLL-AF9 cells. Similar to the results of Hif1 inhibition, this combination therapy improved the survival of leukemia mice (Fig. 8e). Taken together, these data suggest that the therapeutic efficacy of DS-5272 could be enhanced by the simultaneous targeting of Hif1α-PD-L1 pathway.

## Discussion

Pharmacological inhibitors of p53-MDM2 interaction have shown efficacy against several tumors, including acute myeloid leukemia^5,6^. These inhibitors are thought to exert anti-tumor effect by directly inducing cell cycle arrest and apoptosis in tumor cells. Several recent studies have also indicated that p53 may regulate immune responses, especially the CTL response to cancer cells^22^. In our AML models, p53 activation by DS-5272 induced cell cycle arrest, apoptosis and differentiation in AML cells. However, these direct inhibitory effects of DS-5272 on leukemia cells were not sufficient to provide a robust survival benefit for leukemic mice in the absence of antitumor immunity. Importantly, we identified NK cell, but not T-cell, as a critical mediator of therapeutic efficacy of the p53-activating drug against AML. How p53 communicates with NK cells to attack tumor cells and whether p53 is involved in the regulation of innate immune system will merit future research.

The PD-1/PD-L1 checkpoint pathway plays a key role in tumor immune escape^15^. It was previously shown that p53 downregulated PD-L1 through upregulation of miR-34 in non-small cell lung cancer^39^. However, we observed consistent upregulation of PD-L1 in DS-5272-treated MLL-AF9 cells, which is presumably induced by inflammatory cytokines and Hif1α. The activated Hif1α-PD-L1 pathway appears to counteract the NK cell-mediated cytotoxicity, thereby contributes to therapeutic resistance in MLL-AF9 leukemia. Interestingly, upregulation of Hif1α was most evident in differentiated Gr-1+ leukemia cells. These differentiated AML cells could have immunomodulatory functions to increase therapeutic resistance to DS-5272 treatment. Consistent with this observation, the recent single-cell RNA-Seq analyses using AML patient samples revealed that differentiated AML cells express diverse immunomodulatory genes and suppress T cell activity in vitro^40^. Thus, these findings indicate the potential role of differentiated AML cells as immunomodulators to affect disease progression and therapeutic resistance.

Increasing evidence suggests that AML cells remodel the BM microenvironment to support their growth at the expense of normal hematopoiesis^41^. Furthermore, spatial analyses of leukemic bones have revealed that endosteal AML cells produce pro-inflammatory cytokines and are protected from chemotherapy-induced apoptosis^34^. We also found that AML cells in endosteal region express higher level of PD-L1 than those in central marrow and are relatively resistant to p53-induced cell death. Altogether, it appears that endosteal BM regions provide ideal environment to support the survival of AML cells. Factors regulating interaction between leukemia cells and endosteal niche could also be therapeutic targets.

In summary, we show the potent anti-leukemia effect of p53-activating drug against AML, which is markedly enhanced in the presence of NK cells. Our study also provides a rationale for combining p53-activating drugs with Hif1 and/or immune checkpoint inhibitors in the treatment of AML, which warrants further evaluation in clinical trials.

## Methods

### Mice

C57BL/6J mice were purchased from Japan SLC. Ly5.1 mice were maintained in IRCMS, Kumamoto University. NSG mice were purchased from Charles River Laboratories Japan. NSGS mice for PDX experiments were purchased from the Jackson Laboratory. *p53*^*−/−*^ mice, in which 5′ part of exon 2 including translation initiation site of *Trp53* gene was replaced with Neomycin resistance gene, were provided from the RIKEN BioResource Center (Ibaragi, Japan)^42^. *Hif1*α^flox/flox^ mice^43^ were provided from Dr. Randall S. Johnson, and were crossed with Rosa26-Cre-ERT2 mice (Taconic). Rosa26-LSL-Cas9 knockin mice were purchased from Jackson Laboratory^44^. C57BL/6-Tg(CAG-EGFP) mice were purchased from Japan SLC. The primer sequences for genotyping are provided in Supplementary Table 2. All animal studies were approved by the Animal Care Committee of the Institute of Medical Science at the University of Tokyo (approval number: PA13-19, PA15-100, PA17-75), and were conducted in accordance with the Regulation on Animal Experimentation at University of Tokyo based on International Guiding Principles for Biomedical Research Involving Animals.

### Plasmids and viral transduction

We used pMSCV-MLL-AF9-pgk-EGFP or pMSCV-MLL-AF9-pgk-puro for MLL-AF9 expression^45^. Retroviruses were produced by transient transfection of Plat-E packaging cells with retroviral constructs using the calcium-phosphate method, as described previously^46^. Lentiviruses were produced by transient transfection of 293T cells with viral plasmids along with gag-, pol-, and env-expressing plasmids [(pMD2.G #12259) and (psPAX #12260)] using the calcium-phosphate method^47^. Retrovirus transduction to the cells was performed using Retronectin (Takara Bio Inc, Otsu, Shiga, Japan).

### PD-L1 depletion using CRISPR/Cas9

To generate sgRNA expression vectors targeting mouse PD-L1, annealed oligonucleotides were cloned into the lentiGuide-Puro vector, which was obtained from Addgene (plasmid #52963). The expression vector for Cas9 (lentiCas9-Blast #52962) was also obtained from Addgene. MLL-AF9-transduced bone marrow cells were infected with the virus for 24 h, and were selected for stable expression of Cas9 using blasticidin (10 μg/ml) and for stable expression of sgRNAs using puromycin (1 μg/ml) in M3234 (STEMCELL Technologies) methylcellulose containing 20 ng/ml SCF, 10 ng/ml GM-CSF, 10 ng/ml IL-3, and 10 ng/ml IL-6. Sequences for the sgRNAs targeting mouse PD-L1 and Trp53 are provided in Supplementary Table 2.

### Mouse model for AML driven by MLL-AF9

Mouse bone marrow progenitor cells (c-Kit+ cells) derived from wild-type or *Trp*53(−/−) mice were transduced with MLL-AF9, and were transplanted intravenously into sublethally irradiated (525 cGy) recipient mice. MLL-AF9-expressing leukemia cells were harvested from spleens of moribund mice, and were serially transplanted into recipient mice. The serial transplantation was subsequently repeated several times to generate MLL-AF9 cells with strong leukemogenicity. These wild-type and *Trp*53(−/−) MLL-AF9 cells (5 × 10^5^ cells/body) were injected intravenously into non-irradiated recipient mice. For drug studies, DS-5272 (Daiichi Sankyo) was dissolved in 0.5 w/v% Methyl Cellulose 400 Solution (Wako, Japan), and 80 mg/kg/day of DS-5272 was orally administered to mice. Anti-NK1.1 antibody (clone PK136) was dissolved in pH7.0 dilution buffer, and 2.5 mg/kg/day of anti-NK1.1 antibody was injected intraperitoneally once a week. Echinomycin was dissolved in PBS, and 80 μg/kg/day of Echinomycin was injected intraperitoneally. Anti-PD-1 and anti-PD-L1 antibodies were dissolved in pH7.0 and pH6.5 dilution buffers respectively, and 10 mg/kg/day of anti-PD-1 and anti-PD-L1 antibodies were injected intraperitoneally. For long term treatment, DS-5272 was administered on an every 2-day schedule from day 3 to day 13, or from day 11 to 21. Echinomycin was administered 5-days on/ 2-days off schedule from day3 to day14. Anti-PD-1 and anti-PD-L1 antibodies were administered on an every 3-day schedule from day 0 to day 18. For NK cells transfer experiment, NK1.1^+^Cd3^–^ NK cells were collected from spleens of wild-type C57BL/6J mice, and 2 × 10^5^ NK cells were injected intravenously into a leukemia mouse once a week.

### PDX of AML

Proper informed consent was obtained and all experiments were performed according to an institutional review board-approved protocol, in accordance with the Declaration of Helsinki, and with an approved animal study IACUC protocol at CCHMC. Residual diagnostic specimens from AML patients at Cincinnati Children’s Hospital Medical Center were treated with OKT3 antibody and engrafted into NSGS mice^48^. Sensitivity of these PDX-derived AML cells were assessed in IMDM media containing 20% FBS (StemCell Technologies) and 10 ng/mL human SCF, TPO, FLT3L, IL-3, IL-6 with/without DS-5272. The PDX-derived AML cells were plated with titrating doses of DS-5272 (ranging from 0 μM to 2 μM) in triplicate. After 3, 5 or 6 days, 10 μl WST-1 (MK 400; Takara Bio Inc.) or 20 μl MTS (G5440 Promega) cell proliferation assay premix was added to each well. Plates were read at 450–560 nm or 490 nm to measure optical density.

For in vivo treatment studies, 2 million secondary splenocytes from the DS-5272-sensitive PDX-derived AML model [AML-(6)] were transplanted into non-conditioned NSGS mice, and these mice were treated with vehicle (PBS/5%Tween-80/5%PEG-400) or DS-5272 (50 mg/kg, oral gavage) with 5-days on/ 2-days off schedule from day3 to day14. Engraftment of human AML cells was assessed by bone marrow aspiration at day 20 followed by flow cytometry to measure the percentage of human CD45 + CD34 + cells.

### Flow cytometry

Bone marrow cells were obtained by either crushing or flushing femurs and tibias in PBS containing 2% FBS^33^. Red blood cells were removed using RBC lysis buffer. Cells were then stained by fluoro-conjugated antibodies for 30 min at 4°C. After staining, cells were washed with cold PBS for several times, and were resuspended with PBS containing 2% FBS. Cells were analyzed on a FACS Calibur or a FACS Verse, and were sorted with a FACSAria (BD Biosciences, San Jose, CA, USA). Cell cycle analysis (Vybrant DyeCycle Violet stain; ThermoFisher Scientific, Cat#V35003, 1:1000) and apoptosis analysis (Annexin V-APC kit; BD Biosciences, Cat#550475, 1:100) were performed according to the manufacturer’s recommendations. The data were analyzed using FlowJo software (Treestar, Inc., San Carlos, CA). Antibodies used in this study are provided as follows: Brilliant Violet 605TM Streptavidin (BioLegend, San Diego, CA, USA, Cat#405229, 1:400), PE/Cy7-conjugated anti-mouse c-Kit (Clone: 2B8, BioLegend, San Diego, CA, USA, Cat#105813, 1:400), APC-conjugated anti-mouse CD274(Clone:10 F.9G2, BioLegend, San Diego, CA, USA, Cat#124311, 1:400), Gr-1(Clone:RB6-8C5, eBioscience, San Diego, CA, USA, Cat#13-5931-82, 1:400), Mac-1(Clone:M1/70, BioLegend, San Diego, CA, USA, Cat#101203, 1:400), PE-conjugated anti-mouse CD3e(Clone:145-2C11, eBioscience, San Diego, CA, USA, Cat#A14714, 1:400), APC-conjugated anti-mouse CD107a(Clone:1D4B, BioLegend, San Diego, CA, USA, Cat#121613, 1:400), APC-conjugated anti-mouse IFNγ(Clone: XMG1.2, BioLegend, San Diego, CA, USA, Cat#505809, 1:100), Biotin-conjugated anti-mouse NK1.1(Clone: PK136, BioLegend, eBioscience, CA, USA, Cat#13-5941-82, 1:200), PE-Cy^TM^7 Streptavidin (BD Pharmingen^TM^ San Diego, CA, USA, Cat#557598, 1:400), PE-conjugated anti-mouse CD150(Clone:TC15-12F12.2, BioLegend, San Diego, CA, USA, Cat#115903, 1:100), and Brilliant Violet 421TM anti-mouse CD48(Clone:HM48-1, BioLegend, San Diego, CA, USA, Cat#103427, 1:200). Sequential gating/sorting strategies are provided in Supplementary Fig. 13.

### Morphological analysis

Cytospin preparations were stained with Giemsa. Images were obtained with a BX51 microscope and a DP12 camera (Olympus).

### Histological analysis

To enable easy detection of leukemic cells in bone marrow, we transduced MLL-AF9 into bone marrow progenitor cells derived from C57BL/6-Tg (CAG-EGFP), and transplanted MLL-AF9-transduced GFP+ cells into recipient mice. GFP+ leukemia cells were harvested from spleens of moribund mice, and were serially transplanted into recipient mice. The serial transplantation was subsequently repeated several times. The GFP+ MLL-AF9 cells (5 × 10^5^ cells/body) were injected intravenously into non-irradiated recipient mice, following the treatment with vehicle or DS-5272. Femurs were collected 24 h after the treatment, fixed with 4% paraformaldehyde in PBS, decalcified with EDTA, embedded in SCEM (Super Cryoembedding Medium) compound (SECTION-LAB, Japan), and sectioned at a thickness of 7 μm with CM3050 S cryostat (Leica Microsystems, Tokyo, Japan). Sections were analyzed using a microscope (Evos FL Auto 2, Thermo Fisher Scientific).

### Analyses of spatial distribution of AML cells in bone

The epiphyseal and metaphyseal regions of femurs and tibias were removed using the scalpel blade, and were stored in PBS. BM cells in central portion of these bones were flushed out using 21- or 23-gauge needle attach to a 1-ml syringe containing PBS. The flushed bones containing the trabecular region and the epiphyseal and metaphyseal regions of bones were mixed, and were grinded with the pestle. The cells and bone solution were mixed, and was filtered through a 40-mm nylon cell strainer. The frequency of GFP+ cells (=leukemia cells) were assessed using these central and endosteal BM cells collected from the same recipient mice with flow cytometry.

### Western blotting

Cells were lysed in lysis buffer containing 20 mM Tris-HCl (pH 7.4), 137 mM NaCl, 10% glycerol, 2 mM sodium orthovanadate, 2 mM PMSF, 50 mM sodium fluoride and 1% Nonidet P-40. Cell lysates were subject to immunoblotting using the following antibodies: anti-Trp53 (clone 1C12; Cell Signaling Technology, Beverly, MA, Cat#2524, 1:1000), anti-Hif1α: (clone D1S7W; Cell Signaling Technology, Beverly, MA, Cat#36169, 1:1000), and anti-Tubulin (clone B-5-1-2; Santa Cruz Biotechnology Santa Cruz, CA, Cat#sc-23948, 1:5000). Samples were separated by SDS-PAGE and transferred to polyvinylidene difluoride membrane (Millipore Corp., Bedford, MA). The immunoblot was detected with enhanced chemiluminescence reagents (Promega) and visualized with imagequant LAS 4000 (Fujifilm Life Science, Roche Diagnostics). Uncropped and unprocessed scans of the blots are provided in Supplementary Fig. 14.

### qRT-PCR

Total RNAs were isolated from cells using the RNeasy isolation mini-kit (Qiagen) and reverse transcribed by using High Capacity cDNA Reverse Transcription Kits (Applied Biosystems). qRT-PCR was performed using a SYBR Premix EX Taq (Takara). Primer sequences used in this study are provided in Supplementary Table 2.

### RNA-seq analysis

Total RNA was extracted using RNeasy Mini Kit (Qiagen), and the quality and quantity of RNA were checked using Agilent High Sensitivity RNA Screen Tape and Qubit. RNA libraries were prepared using 1000 ng total RNA with SureSelect Strand-Specific RNA Preparation Kit (Agilent) according to the manufacturer’s protocol. The quality and quantity of these libraries were checked using Agilent TapeStation D1000 and KAPA Library Quantification Kits [KAPA BioSystems] / Real-time PCR Systems Step One Plus [Applied Biosystems]. These libraries were sequenced on the Illumina HiSeq2500 System with 2 × 100 nucleotide paired-end reads according to the manufacturer’s protocol. Derived reads were processed using cutadapt(1.8.1) and fastx-toolkit(0.0.13) to remove Illumina adaptor sequence and to trim low-quality bases. Quality of reads were assessed using FastQC. Processed reads were aligned to GRCm38 reference transcripts using TopHat(2.1.1)-Cufflinks(2.2.1) pipeline^49–51^ to derive gene FPKM values.

### Single-cell mass cytometry analysis

A summary of all mass cytometry antibodies, reporter isotopes and concentrations used for analysis are provided in Supplemental Table 3. Primary conjugates of mass cytometry antibodies were purchased pre-conjugated from Fluidigm, or prepared using the MaxPAR antibody conjugation kit (Fluidigm PRD002 Version7) according to the manufacturer’s recommended protocol.

For sample preparation, MLL-AF9 cells were isolated from femurs and tibias of mice treated with vehicle or DS-5272 24 h before. After filtration with Falcon Cell Strainer (mesh size 40 μm, Cat# 087711), red cells were removed using 1x ACK buffer (provided by H.T.) These cells were counted and resuspended in PBS (1 × 10^7^ per ml), stained with live/dead cell indicator Cell-ID^TM^ Cisplatin (Fluidigm, Cat# 201198) at a final concentration of 5 μM, and were then fixed with 1 × Maxpar FixIBuffer (Fluidigm, Cat# 201065). 1 × 10^6^ cells/sample were aliquoted and were stained with 26 antibodies (Supplementary Table 3) according to the Maxpar Phospho-protein Staining (Fluidigm, PRD016 Version3) protocol. At the end of cell staining, cells were labeled with Cell-ID^TM^ Intercalator–Ir in Maxpar Cell Staining Buffer (Fluidigm Cat#201068) at 4 °C overnight for DNA intercalation.

Stained samples were washed twice with Maxpar Cell Staining Buffer (Fluidigm Cat#201068) and once with Maxpar Water (Fluidigm, Cat# 201069). Then, cells (2.5 × 10^5^ per ml) in Maxpar Water (Fluidigm, Cat# 201069) were added with EQ^TM^ Four Element Calibration Beads (Fluidigm, Cat#201087) diluted to 1/10. The samples were filtered with 40 μm Cell Strainer Snap Cap Falcon™ Test Tube (Cat#0877123) immediately prior to sample acquisition on Helios^TM^ mass cytometer (Fluidigm, Cat#107002). Approximately 100 K events per sample were acquired for each sample. FCS files were normalized to the *EQ Four Element Calibration Beads* using Helios software *(Version 6.5.358)*. The data (FSC files) were downsampled from 100,000 into 3000 cell events using FlowJo 10.3. and were then analyzed by SPRING (https://kleintools.hms.harvard.edu/tools/spring.html). For SPADE analysis, gating and extraction of median expression levels were performed using Cytobank (https://premium.cytobank.org) under a condition of Target Number of Nodes is 150. The file was downsampled to an absolute number of 5000. The CD45+ population and 8 clustering channels (141Pr_Gr1, 143Nd_CD41,145_Nd_CD4,160Gd_B220.168Er_CD8a, 170Er_CD49d,172Yb_Mac-1,173Yb_ckit) were selected to create categorization for the SPADE.

### Co-culture of MLL-AF9 leukemia cells and NK cells

NK1.1^+^Cd3^-^ NK cells were collected from spleens of wild-type C57BL6/J mice. 5 × 10^4^ MLL-AF9 cells were co-cultured with 1 × 10^4^ NK cells in RPMI media containing 10% FBS, 100 ng/mL mouse IL-2 and 1 ng/mL mouse IL-3. Apoptosis was assessed as described above after 48 h of culture.

### Statistics

Statistical analyses for evaluating differences between two groups were performed by the unpaired and two-tailed Student’s *t* test. The survival distributions were compared by the log-rank test. GraphPad Prism 7 was used for these statistical analyses. No specific statistical methods were used to predetermine the sample size.

### Reporting summary

Further information on research design is available in the Nature Research Reporting Summary linked to this article.

## Supplementary information

Supplementary Information

Description of Additional Supplementary Files

Supplementary movie 1

Supplementary movie 2

Supplementary movie 3

Supplementary movie 4

Reporting Summary

## Data Availability

The RNA-sequencing data have been deposited in the NCBI gene expression omnibus under the accession code GSE135008. The Mass-cytometry data referenced during the study are available in Mendeley Data (10.17632/fsrwv9hpt8.1). All the other data supporting the findings of this study are available within the article and its supplementary information files and from the corresponding author upon reasonable request. A reporting summary for this article is available as a Supplementary Information file.
