# Supplementary material for: Antitumor immunity augments the therapeutic effects of p53 activation on acute myeloid leukemia
Source: Nat Commun. 2019 Oct 25;10:4869. doi: 10.1038/s41467-019-12555-1 (PMC6814808; doi:10.1038/s41467-019-12555-1)
Supplement: Supplementary file 1 — Supplementary Information [file 41467_2019_12555_MOESM1_ESM.pdf]

Supplementary information

Antitumor immunity augments the therapeutic effects of p53 activation on acute myeloid leukemia.

Hayashi et al.

Supplementary Table 1. Information of each human AML sample used in this study.
Viability of cells treated with DS-5272 was measured by WST-1 assay. Results are normalized to the viability of DMSO-treated cells, set at 1(n=3). Data are shown as the average and SD of duplicate wells.

Compound		Concentration (uM)										
DS-5272		2.048	1.024	0.512	0.256	0.128	0.064	0.032	0.016	0.008	0	Genomic Alterations
Sample ID												
AML-(1)	Avg (%Viable)	103%	129%	141%	118%	112%	128%	115%	108%	95%	100%	FLT3 D835E – subclonal # , D835V – subclonal # , D835Y – subclonal # KIT D816V ASXL1 G645fs*58 CBL deletion exon 9 CHEK2 T367fs*15 PTPN11 D61V , inv(3)
	St. Dev	13.2%	25.6%	41.6%	7.7%	12.4%	26.2%	2.8%	1.9%	11.6%	0.8%	
AML-(2)	Avg (%Viable)	95%	106%	94%	92%	94%	96%	94%	100%	92%	100%	CDKN2A/B p16INK4a loss and p14ARF loss exon 1 and CDKN2B loss
	St. Dev	22.1%	8.5%	7.8%	2.6%	13.1%	8.1%	1.3%	8.6%	2.0%	7.8%	
AML-(3)	Avg (%Viable)	54%	77%	85%	92%	101%	113%	99%	99%	105%	100%	KRAS G12A – subclonal # NRAS Q61H – subclonal # KMT2A (MLL) MLL-MLLT4 (AF6) fusion PTPN11 E76K – subclonal # , S502L – subclonal #
	St. Dev	2.6%	6.3%	10.8%	13.0%	12.6%	9.3%	0.3%	26.3%	13.1%	3.3%	
AML-(4)	Avg (%Viable)	2%	2%	17%	48%	76%	79%	84%	90%	98%	100%	t(7;12)(q36;p13) MNX1/ETV6 fusion PTPN11 p.D61V c.182A>T
	St. Dev	0.3%	1.2%	8.6%	15.4%	13.3%	4.1%	7.9%	1.3%	17.7%	6.2%	
AML-(5)	Avg (%Viable)	40%	62%	68%	76%	93%	87%	107%	98%	107%	100%	MLL MLL-MLLT3 (AF9) fusion PC R583H – subclonal # WT1 R462Q – subclonal#
	St. Dev	1.0%	7.1%	4.9%	0.1%	10.9%	17.7%	3.9%	10.8%	11.5%	9.4%	
AML-(6)	Avg (%Viable)	2%	9%	38%	45%	73%	89%	82%	95%	93%	100%	FLT3 D835A – subclonal # , D835E – subclonal # , D835Y –subclonal #, I836del – subclonal#, V491L – subclonal#,V579A – subclonal# KRAS G13D MLL MLL-MVB12B fusion MLLT10 LL-MLLT10 (AF10) fusion
	St. Dev	0.6%	0.0%	0.5%	3.1%	13.5%	1.9%	3.7%	3.7%	1.0%	2.7%	
AML-(7)	Avg (%Viable)	33%	49%	60%	67%	78%	83%	94%	92%	91%	100%	MLL inv(11)(p11.2q23) ; HPIM- β chain regulatory sequence on 7q fusion
	St. Dev	3.2%	0.8%	2.4%	1.0%	5.2%	1.5%	11.1%	6.9%	6.7%	4.9%	
AML-(8)	Avg (%Viable)	7%	18%	31%	49%	70%	70%	86%	96%	90%	100%	CBFA2T3-GLIS2 rearrangement
	St. Dev	1.0%	1.5%	1.9%	3.2%	10.9%	2.5%	0.6%	8.7%	3.8%	4.2%	
AML-(9)	Avg (%Viable)	12%	18%	38%	58%	74%	75%	105%	87%	96%	100%	MLL MLL-MLLT3 (AF9) fusion
	St. Dev	2.2%	3.4%	7.0%	0.1%	2.1%	9.2%	8.3%	3.1%	13.5%	4.4%	
AML-(10)	Avg (%Viable)	127%	124%	106%	112%	108%	106%	105%	101%	104%	100%	NF1 Q1775* PTPN11 A461G – subclonal # CDKN2A/B loss ETV6 loss MLLT10 PICALM-MLLT10 fusion PHF6 R274Q TP53 K164E – subclonal # , Y126*
	St. Dev	11.1%	9.5%	12.4%	3.5%	3.9%	10.7%	11.4%	3.5%	6.2%	9.7%	
AML-(11)	Avg (%Viable)	17%	29%	41%	53%	72%	83%	89%	97%	95%	100%	DNMT3A R882C FLT3 L576_Q577ins17 PTPN11 D61Y NPM1 W288fs*10+ WT1 A382fs*11, A382fs*4
	St. Dev	1.6%	3.9%	3.9%	0.7%	1.7%	2.9%	9.8%	2.0%	5.0%	0.7%	

Supplemental Table 2. Sequences of primers used in this study.

Name	Forward (5'-3')	Reverse (5'-3')	Application
Cdkn1a	ATCACCAGGATTGGACATGG	CGGTGTCAGAGTCTAGGGGA	qRT-PCR
Mdm2	CTGCTCTCACTCAGCGATGT	TCTGTGAAGGAGCACAGGAA	qRT-PCR
Bbc3	TGTCGATGCTGCTCTTCTTG	GTGTGGAGGAGGAGGAGTGG	qRT-PCR
Gadd45a	AGACCGGAAAGGATGGACAC	GTACACGCCGACCGTAATG	qRT-PCR
Gapdh	TGTGTCCGTCGTCGTGGATCTGA	TTGCTGTTGAAGTCGCAGGAG	qRT-PCR
Trp53 WT	GTTATGCATCCATACAGTACA	CCGCAGGATTTACAGACACC	Genotyping
Trp53 KO	ACGTGGTTGGTTACCTCTGC	TCGCCTTCTTGACGAGTTCT	Genotyping
Hif1a	AGATAGGAAGATCTCCCAGTTCAGT	GAAAACTGTCTGTAACTTCATTTCC	Genotyping
Cre-ERT2	TCGATGCAACGAGTGATGAG	TTCGGCTATACGTAACAGGG	Genotyping
NT	caccgCGCTTCCGCGGCCCGTTCAA	aaacTTGAACGGGCCGCGGAAGCGc	sgRNA
sgTrp53	caccgGAAGTCACAGCACATGACGG	aaacCCGTCATGTGCTGTGACTTCc	sgRNA
sgPD-L1	caccgGCCTGCTGTCACTTGCTACG	aaacCGTAGCAAGTGACAGCAGGCc	sgRNA

Supplemental Table 3. Antibodies used for Mass Cytometry analysis.

Metal label	Specificity	Clone	Vendor	Cat No.
141Pr	Ly-6G/C(Gr1)	RB6-8C5	Fluidigm	3141005B
143Nd	CD41	MWreg30	Fluidigm	3143009B
145Nd	CD4	RM4-5	Fluidigm	3145002B
146Nd	VCAM1	429 (MVCAM.A)	BioLegend	105702
147Sm	CD45.2	104	Fluidigm	3147004B
149Sm	CXCR4(CD184)	L276F12	Biolegend	146502
150Nd	HIF1α	H206	Santa Claus	sc-10790
151Eu	CD49d (Integrin alpha 4)	R1-2	Fluidigm	3151016B
152Sm	phospho-Akt [S473]	D9E	Fluidigm	3152005A
153Eu	phospho-STAT1 [Y701]	58D6	Fluidigm	3153003A
156Gd	phospho-p38 MAP Kinase [T180/Y182]	D3F9	Fluidigm	3156002A
158Gd	phospho-STAT3 [Y705]	4/P-STAT3	Fluidigm	3158005A
159Tb	phospho-MAPKAP Kinase2 [T334]	27B7	Fluidigm	3159010A
160Gd	CD45R (B220)	RA3-6B2	Fluidigm	3160012B
161Dy	Ki-67	B56	Fluidigm	3161007B
162Dy	p53 (For whole protein)	X77	Abcam	ab16465
163Dy	phospho-mTOR [S2448]	EPR426(2)	Abcam	ab109268
165Ho	beta-Catenin	D13A1	Fluidigm	3165027A
167Er	EPCR（CD201)	eBio1560 (1560)	eBioscience	16-2012-83
168Er	CD8a	53-6.7	Fluidigm	3168003B
169Tm	Ly-6A/E (Sca-1)	D7	Fluidigm	3169015B
170Er	CD49b	HMa2	Fluidigm	3170008B
171Yb	phospho-ERK 1/2 [T202/Y204]	D13.14.4E	Fluidigm	3171010A
172Yb	CD11b (Mac-1)	M1/70	Fluidigm	3172012B
173Yb	CD117 (ckit)	2B8	Fluidigm	3173004B
174Yb	phospho-STAT4	38/p-Stat4	Fluidigm	3174005A
175Lu	PGC1 alpha	polyclonal	Abcam	ab54481
191Ir	Cell-ID^{TM} Intercalator–Ir (DNA staining)	NA	Fluidigm	201192A
193Ir	Cell-ID^{TM} Intercalator–Ir (DNA staining)	NA	Fluidigm	201192A
198Pt	Cell-ID^{TM}Cisplatin (Live/Dead Cell staining)	NA	Fluidigm	201198
NA: not available				

Supplementary Figure 1

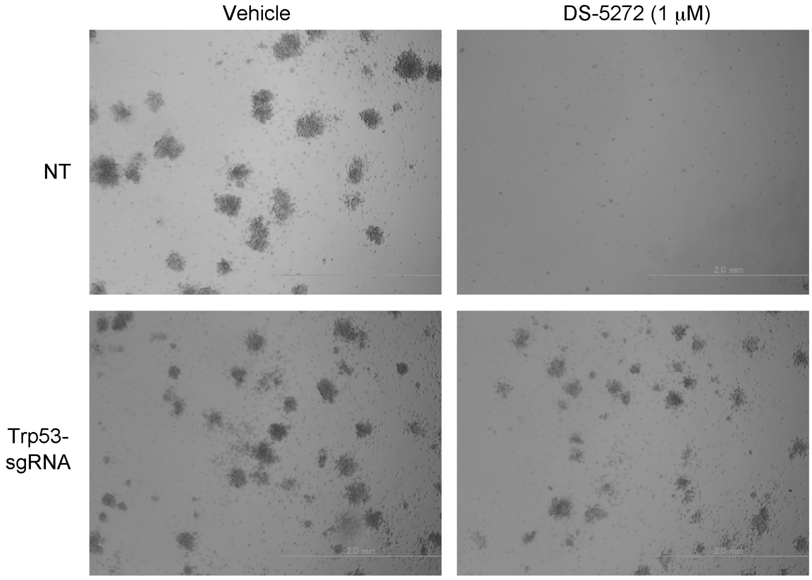

Supplementary Figure 1. p53-deficient MLL-AF9 cells are resistant to DS-5272.

5 x 10^4 MLL-AF9 cells, transduced with Cas9 together with non-targeting (NT) or Trp53-targeting sgRNA, were plated in MethoCult™ M3234 supplemented with 10 ng/ml mouse SCF, 10 ng/ml, mouse GM-CSF, 10 ng/ml mouse IL-3, and 10 ng/ml mouse IL-6, with/without DS-5272 (1 mM) for 5 days. Representative photos of each plate are shown (Scale bars: 2 mm).

Supplementary Figure 2

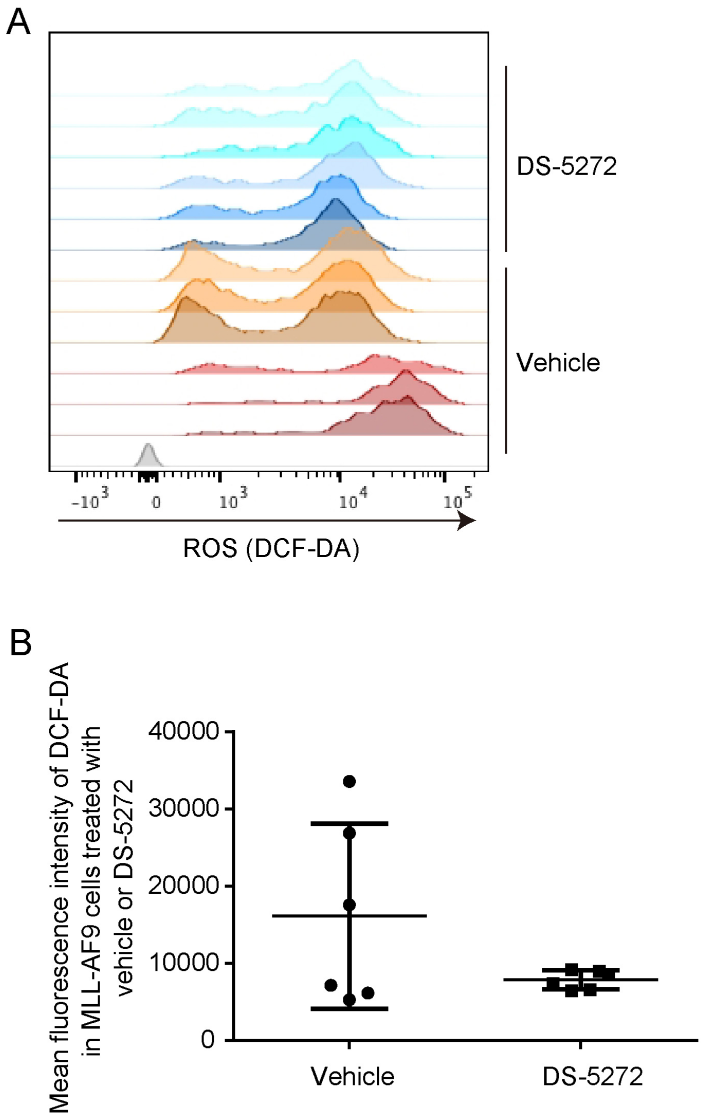

Supplementary Figure 2. DS-5272 does not induce ROS overproduction in MLL-AF9 cells. **A.** FACS plots showing intensity of ROS (DCF-DA) in MLL-AF9 leukemia cells. Leukemia cells were isolated from bone marrow 24 hours after treatment with vehicle control or DS-5272. **B**. Quantification of MFI of ROS (DCF-DA) in MLL-AF9 leukemia cells. Data are shown as mean ± s.d.

Supplementary Figure 3

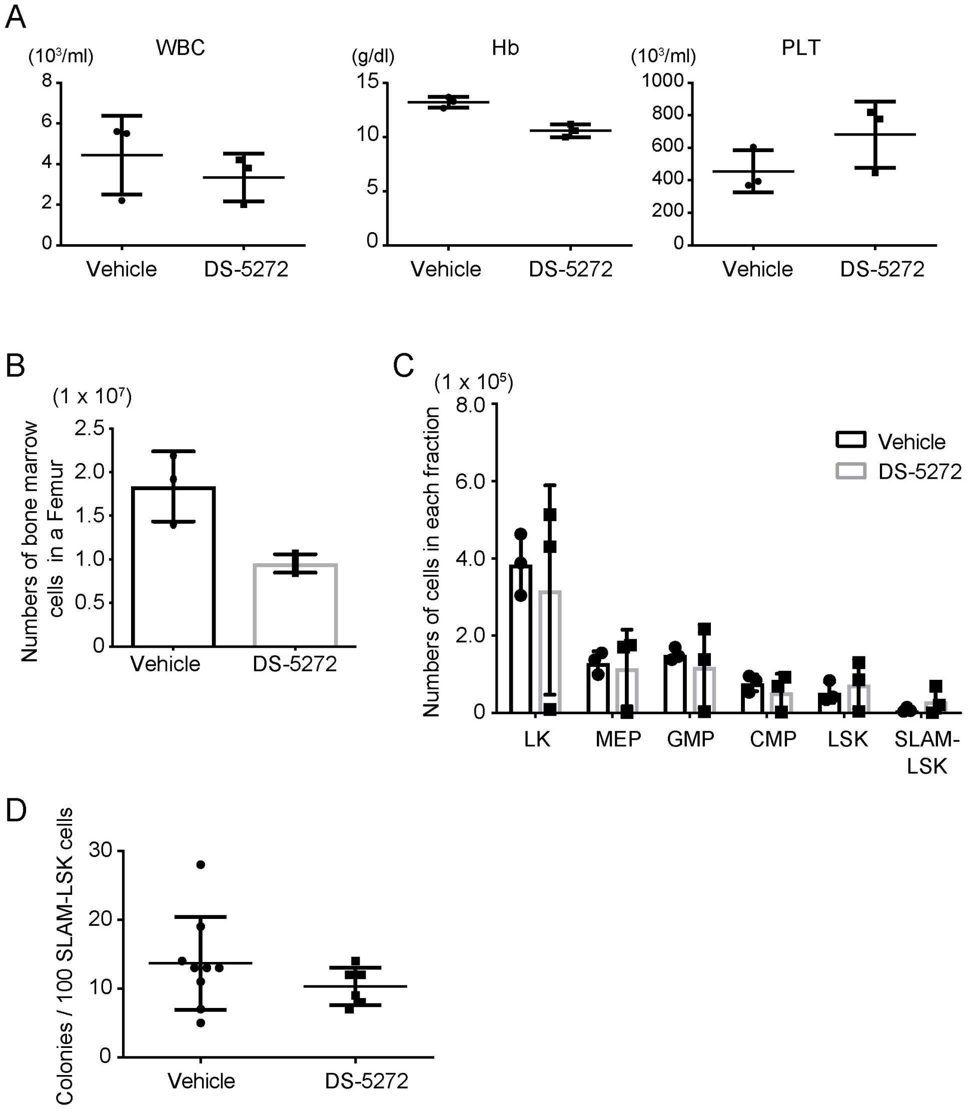

Supplementary Figure 3. DS-5272 does not significantly inhibit normal hematopoiesis.

Female 8-weeks-old C57BL/6 mice were treated with vehicle or DS-5272 for 2 weeks (80mg/kg, 3 times per week). (**A-C**). Shown are WBC counts, Hb levels and PLT counts (**A**), numbers of bone marrow cells in a femur (**B**), and numbers of hematopoietic stem and progenitor cells (**C**) in mice treated with vehicle or DS-5272. **D**. 100 SLAM-LSK cells were sorted and were used for colony forming assay. LK: lineage$^-$cKit$^+$, MEP: megakaryocyte/erythroid progenitor, GMP: granulocyte/monocyte progenitor, CMP: common myeloid progenitor, LSK: lineage$^-$Sca1$^+$cKit$^+$, SLAM-LSK: CD150$^+$CD48$^-$lineage$^-$Sca1$^+$cKit$^+$. Data are shown as mean ± s.d.

Supplementary Figure 4

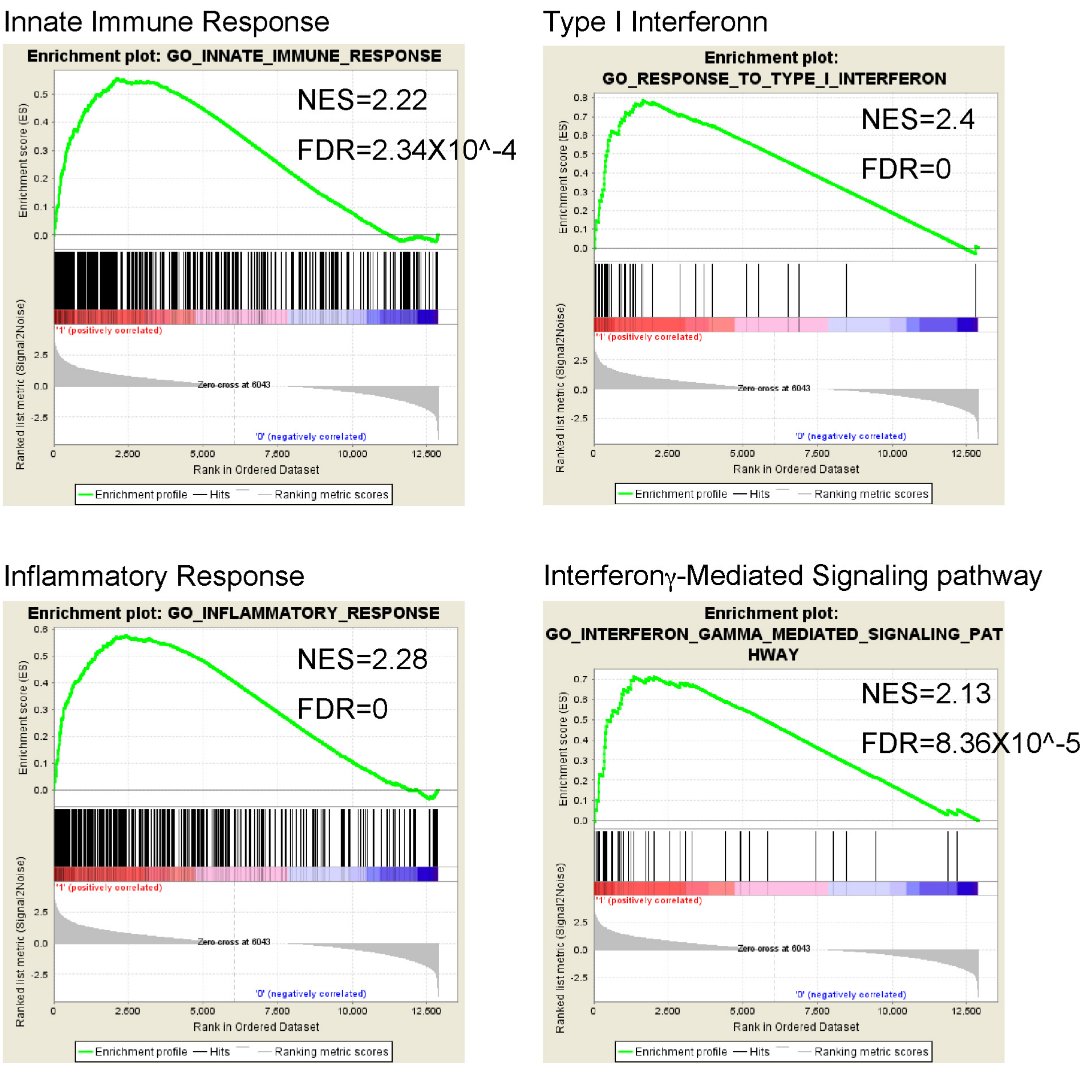

Supplementary Figure 4. GSEA of upregulated genes in MLL-AF9 cells treated with DS-5272. GSEA revealed that genes related to Innate Immune Response, Type 1 Interferon, Inflammatory Response, and Interferon-γ-Mediated Signaling Pathway were upregulated in MLL-AF9 cells treated with DS-5272 compared with those treated with vehicle.

Supplementary Figure 5

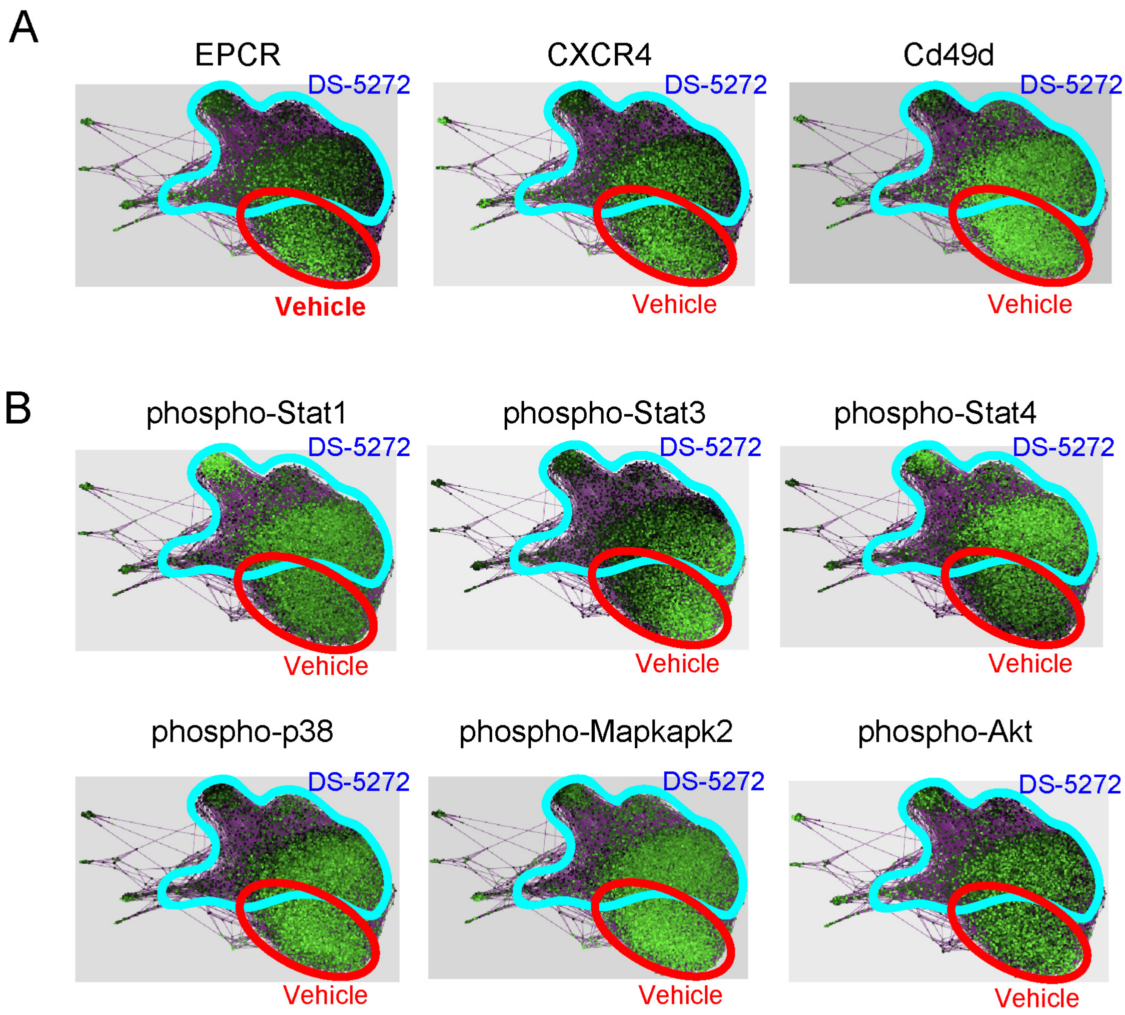

Supplementary Figure 5. SPRING plots of MLL-AF9 cells treated with Vehicle or DS-5272. **A**, **B**. 25-dimensional analysis using SPRING (https://kleintools.hms.harvard.edu/tools/spring.html) of bone marrow cells from MLL-AF9 leukemia mice treated with vehicle and DS-5272 for 24 hours (n=2/condition). Differences in the location of cells within the SPRING map resulted from changes in protein expression. The regions enclosed by the red and blue curves represent the cells treated with vehicle or DS-5272, respectively. Cell surface expression of adhesion molecules (EPCR, CXCR, Cd49d, **A**) and intracellular signaling pathway markers (phospho-Stat1, phospho-Stat3, phospho-Stat4, phospho-p38, phospho-Mapkapk2, phosphor-Akt, **B**) in vehicle- and DS-5272-treated MLL-AF9 leukemia cells are shown.

Supplementary Figure 6

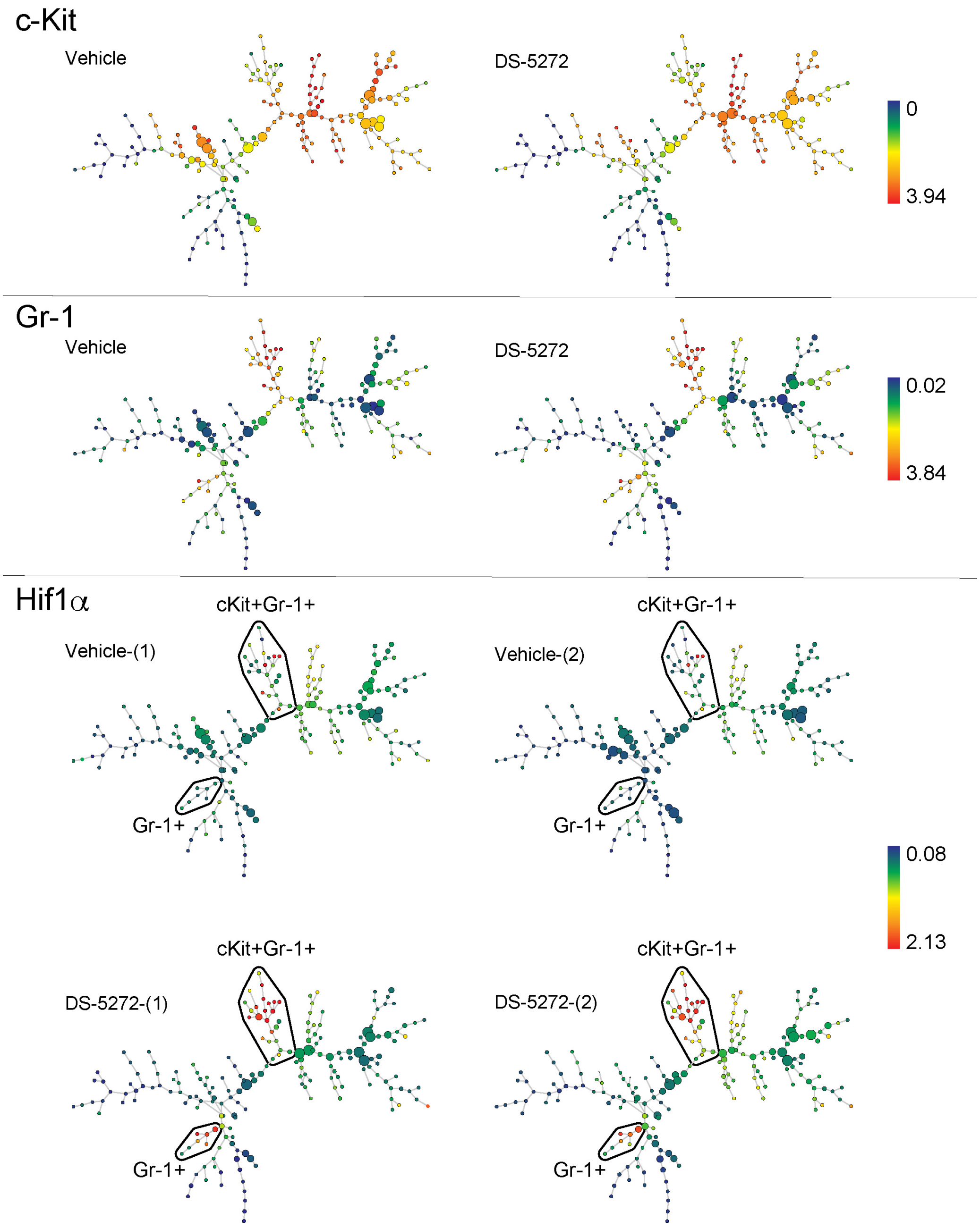

Supplementary Figure 6. SPADE plots of MLL-AF9 cells treated with Vehicle or DS-5272.

SPADE plots of bone marrow cells from MLL-AF9 leukemia mice treated with vehicle or DS-5272 for 24 hours (n=2/condition). MLL-AF9 leukemia cells were grouped based on cell surface expression of c-Kit and Gr-1. Protein expression of HIF1α in vehicle- and DS-5272-treated MLL-AF9 leukemia cells are shown.

Supplementary Figure 7

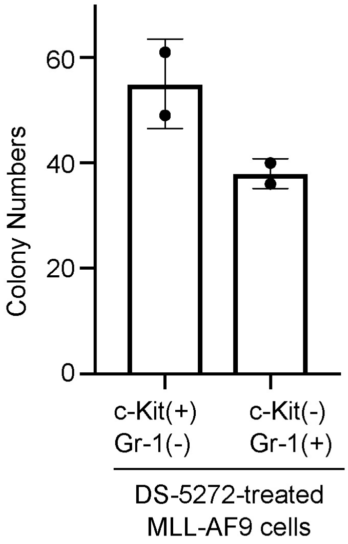

Supplementary Figure 7. Gr-1+ MLL-AF9 cells retain clonogenic activity.

MLL-AF9 leukemia mice were treated with DS-5272, and spleen cells were collected from them 24 hours after the treatment. $GFP^{+}c\text{-}Kit^{+}Gr\text{-}1^{-}$ cells and $GFP^{+}c\text{-}Kit^{-}Gr\text{-}1^{+}$ cells were isolated by fluorescence-activated cell sorting and colony formation assays were performed in duplicates. Data are shown as mean ± s.d.

Supplementary Figure 8

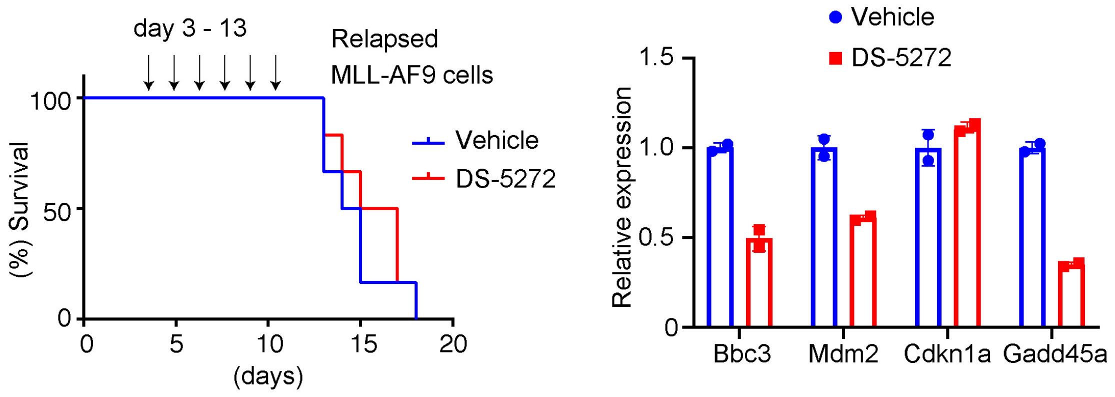

Supplementary Figure 8. Relapsed MLL-AF9 cells are resistant to DS-5272.

A. MLL-AF9 cells were collected from mice that became moribund after DS-5272 treatment, and were serially transplanted into recipient mice. The mice injected with the relapsed MLL-AF9 leukemia cells after DS-5272 treatment were again treated with vehicle or DS-5272 from day 3 to day 13. Kaplan-Meier survival curves of these mice are shown. DS-5272 showed no inhibitory effects on the relapsed MLL-AF9 leukemia cells. **B**. GFP+ MLL-AF9 leukemia cells were collected from bone marrows of vehicle- or DS-5272-treated mice. Expression levels of p53-target genes in MLL-AF9 cells were assessed by qPCR. The relapsed MLL-AF9 cells were no longer responsive to DS-5272 treatment. Data are shown as mean ± s.d.

Supplementary Figure 9

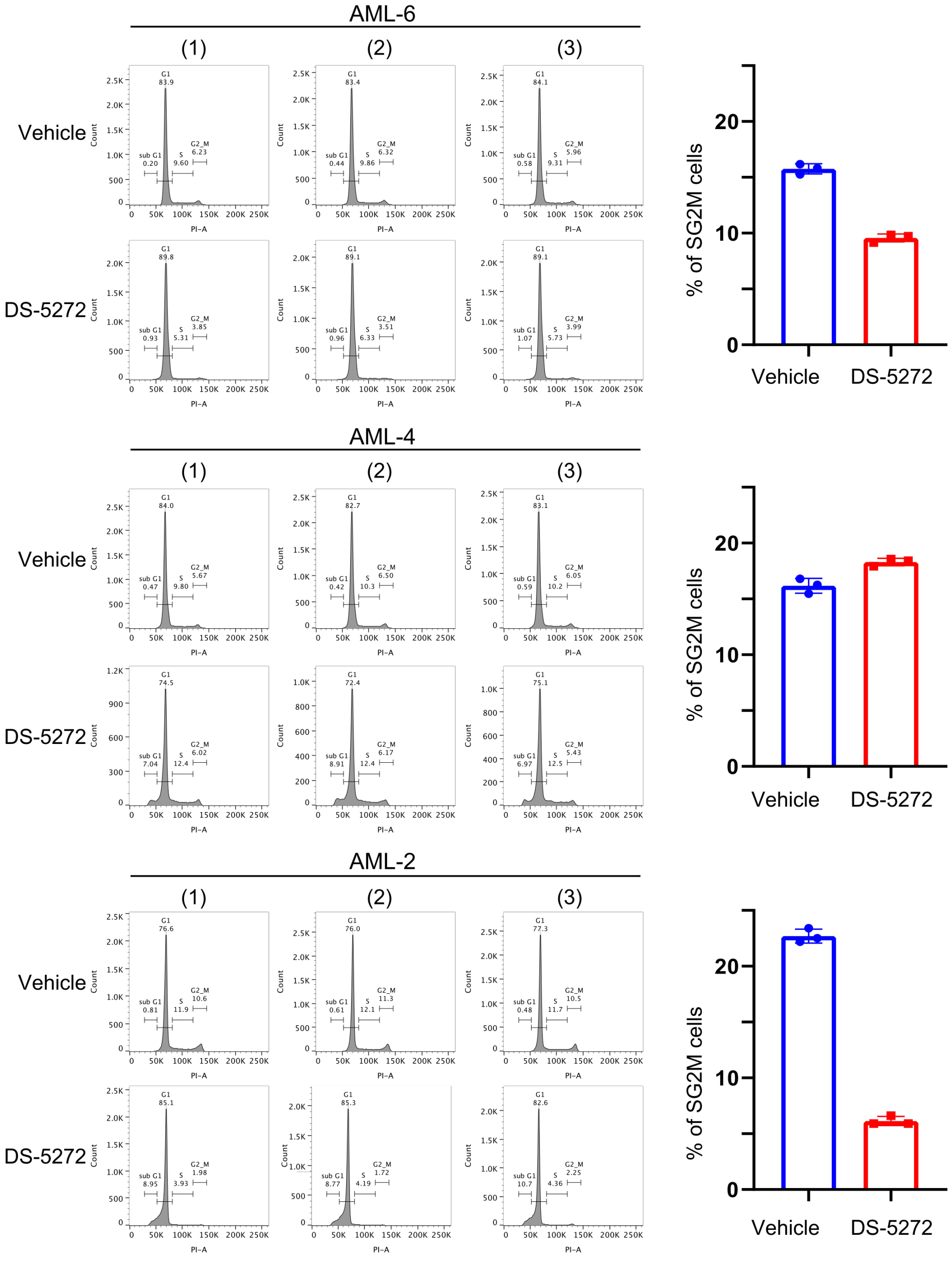

Supplementary Figure 9. Cell-cycle status of human AML cells treated with DS-5272. Cell-cycle status was assessed after 3 days culture with vehicle or DS-5272 (250 nM) using human AML-(2), (4) and (6) cells. Shown are cell cycle profiles assessed by FACS with PI staining of DNA. The numbers indicate the percentages of cells in the sub-G1, G1, S and G2/M phases. See also Figure 6B.   Data are shown as mean ± s.d.

Supplementary Figure 10

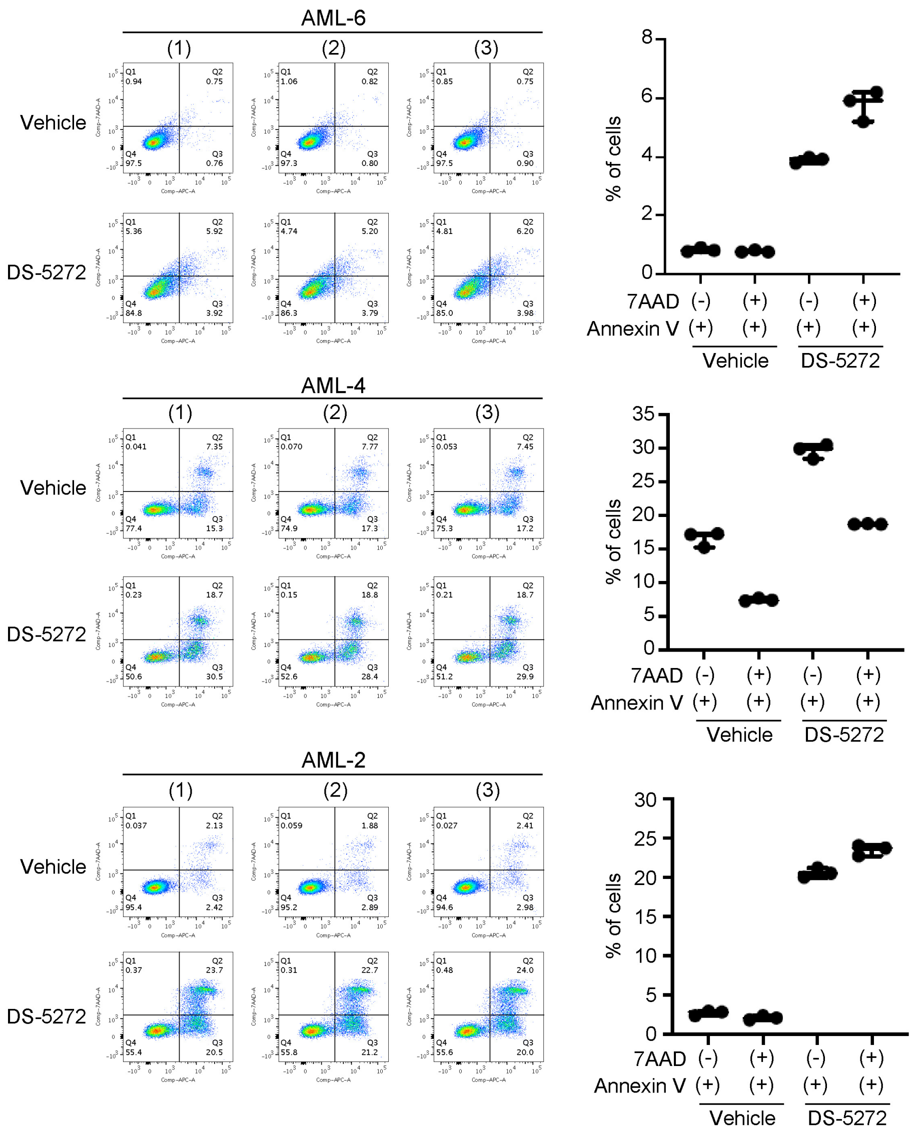

Supplementary Figure 10. Apoptosis of human AML cells treated with DS-5272.

Apoptosis was assessed after 3 days culture with vehicle or DS-5272 (250 nM) using human AML-(2), (4) and (6) cells. Shown are FACS profiles of Annexin V and PI staining. The numbers indicate the percentages of Annexin V+ cells. See also Figure 6B. Data are shown as mean ± s.d.

Supplementary Figure 11

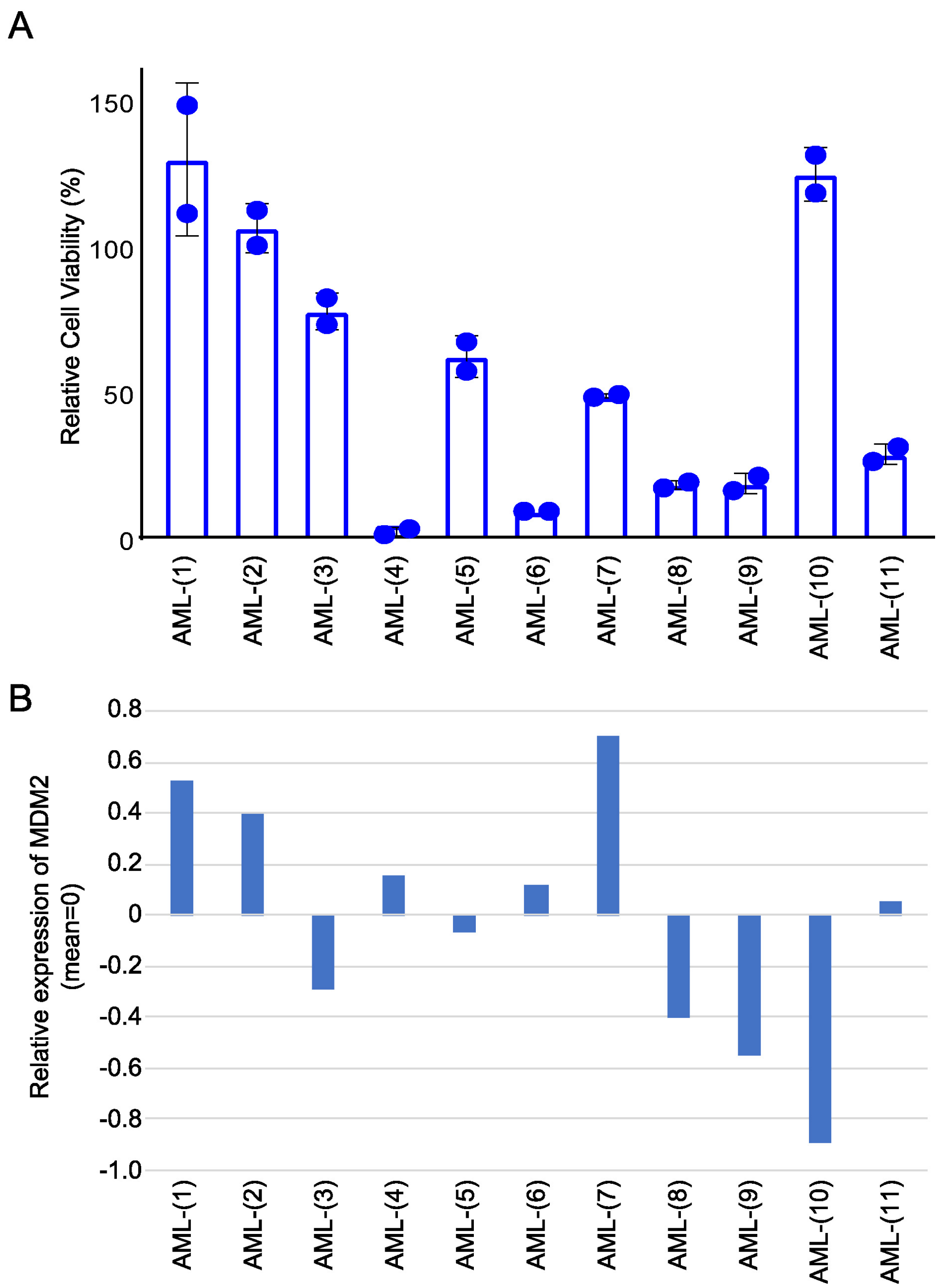

Supplementary Figure 11. MDM2 expression does not predict the sensitivity of MLL-AF9 cells to DS-5272.

A.Viability of eleven PDX-derived human AML cultures treated with DS-5272 (1 μM) was measured by MTS assay. Results are normalized to the viability of DMSO-treated cells, set at 1 (n = 3). AML-(10) has a TP53 mutation. Data are shown as the mean ± SD of triplicate wells. **B**. Relative MDM2 levels in the eleven PDX-derived human AML cells. See also Figure 6A and Supplementary Table 1.

Supplementary Figure 12

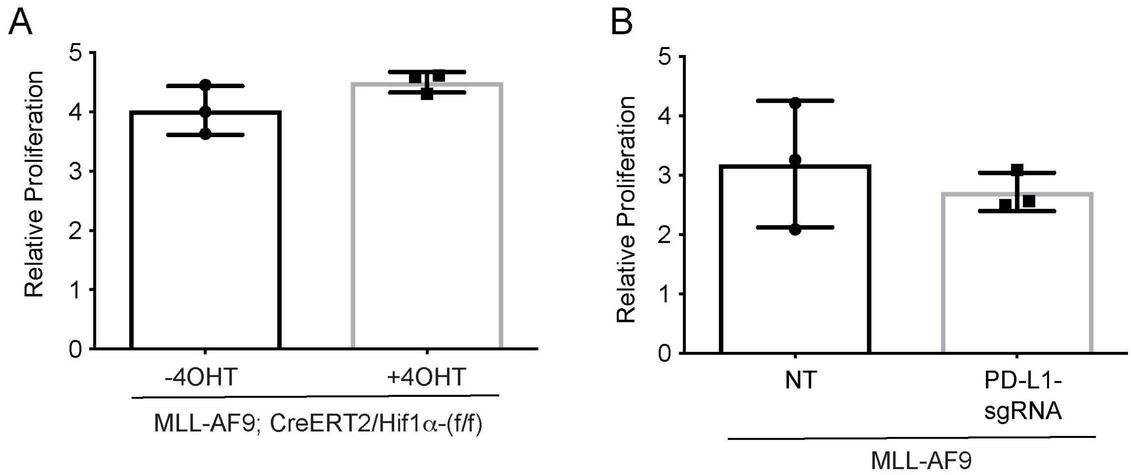

Supplementary Figure 12. Depletion of PD-L1 or Hif1a does not alter the growth of MLL-AF9 cells.

A, B. Effects of HIF1α (**A**) or PD-L1 (**B**) depletion on the growth of MLL-AF9 cells were measured by WST-8 assay. Depletion of these genes did not change the *in vitro* growth of MLL-AF9 cells. Data are shown as mean ± s.d.

Supplementary Figure 13

A. Gating strategy for MLL-AF9 cells, NK cells and Cd8+ T cells.

B. Gating strategy for SLAM-LSK, CMP, GMP and MEP cells.

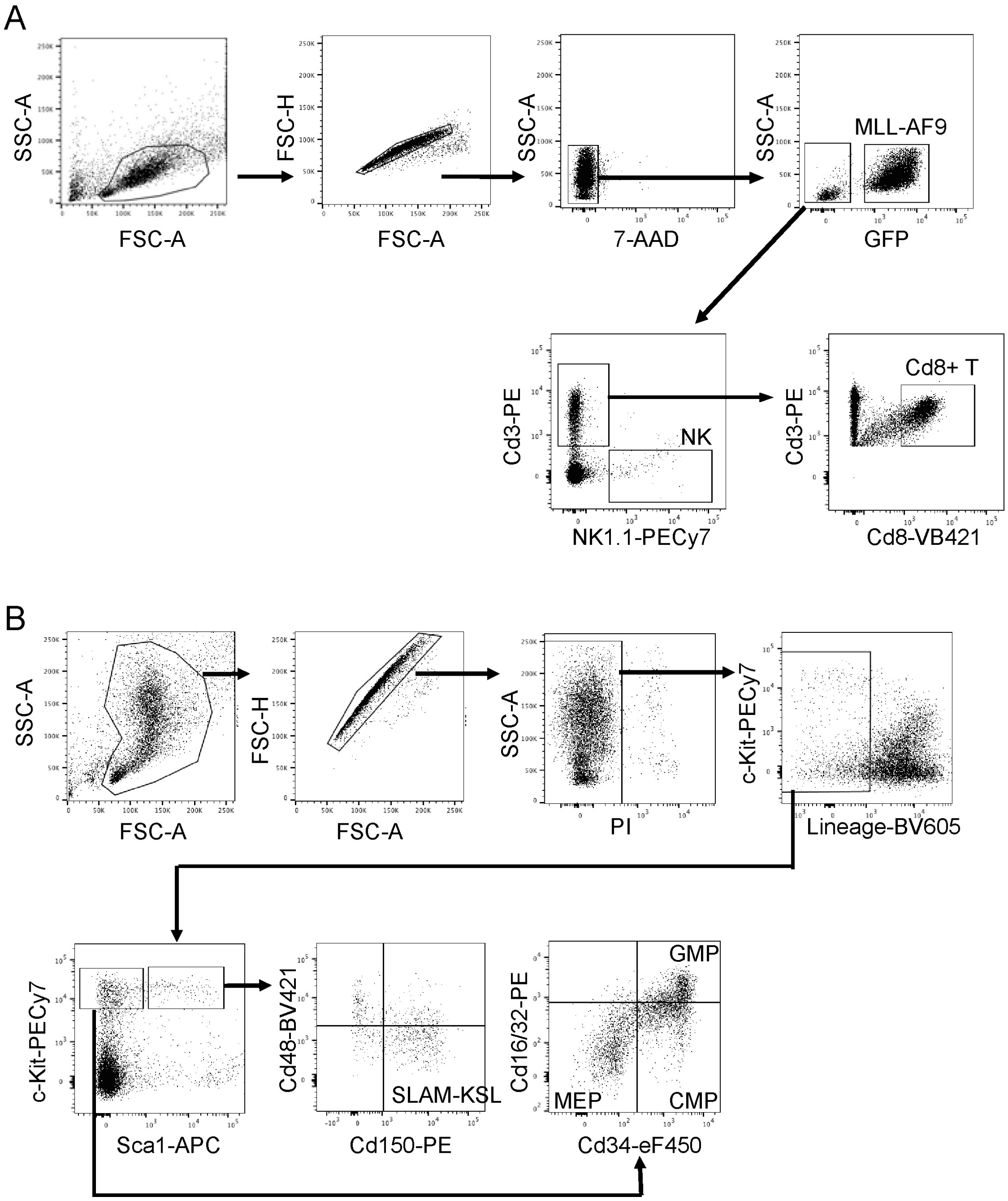

Supplementary Figure 13. Gating strategies used for flow cytometry.

Supplementary Figure 14

A. Full blots for Figure 1C.

B. Full blots for Figure 4B.

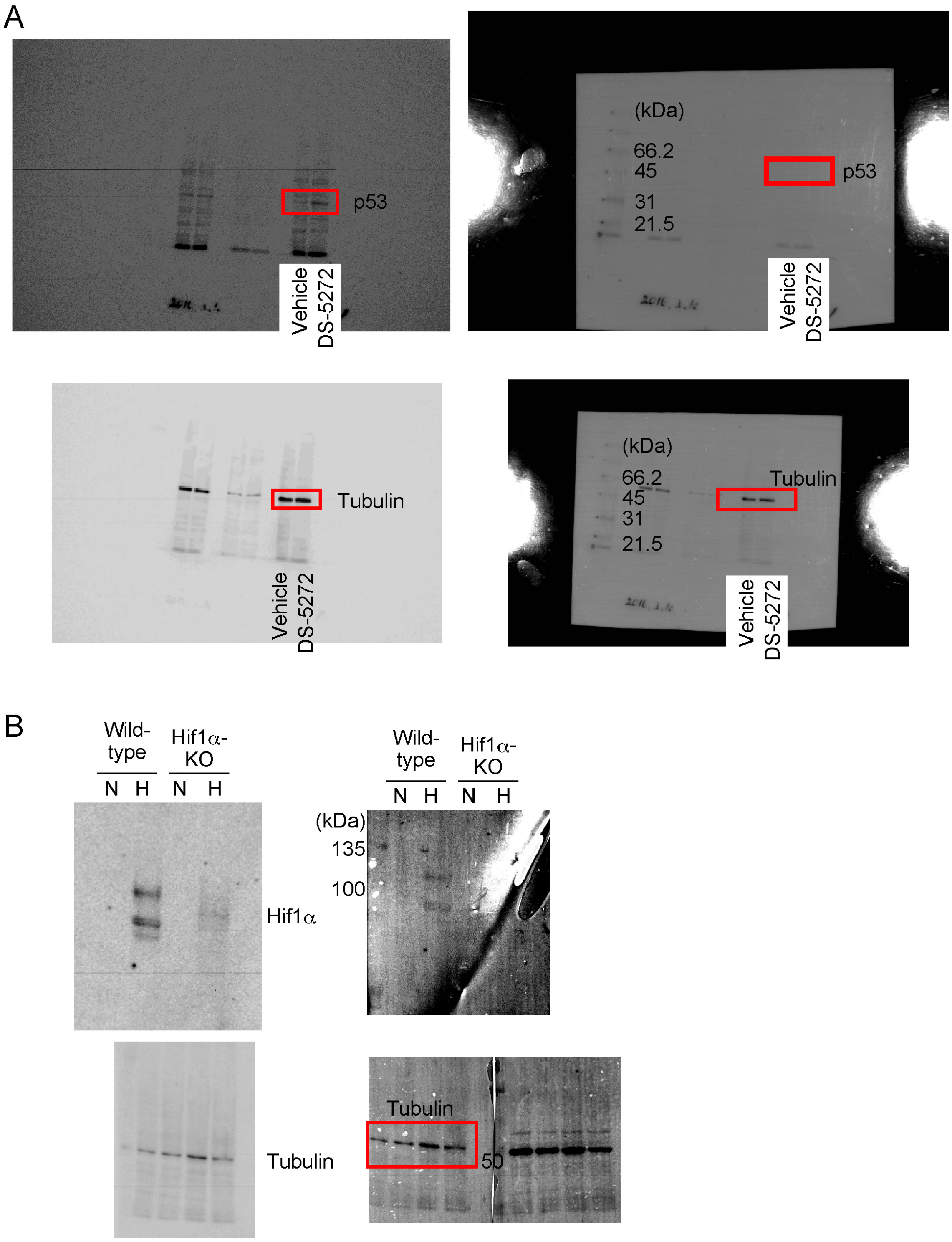

Supplementary Figure 14. Uncropped immunoblot images of Figure 1C and 4B.
