# Supplementary material for: Antitumor immunity augments the therapeutic effects of p53 activation on acute myeloid leukemia
Source: Nat Commun. 2019 Oct 25;10:4869. doi: 10.1038/s41467-019-12555-1 (PMC6814808; doi:10.1038/s41467-019-12555-1)
Supplement: Supplementary file 2 — Description of Additional Supplementary Files [file 41467_2019_12555_MOESM2_ESM.docx]

Description of Additional Supplementary Files

**Supplementary movies 1, 2** (Dataset video before1, 2) The MLL-AF9 leukemia mouse developed hind limb paralysis.

**Supplementary movies 3,4** (Dataset video after1, 2) The mouse shown in supplementary movie 1 and 2 was treated with DS-5272. Twenty-four hours after the treatment, the mouse no longer showed hind limb paralysis and started to walk actively.
