# Supplementary material for: Antitumor immunity augments the therapeutic effects of p53 activation on acute myeloid leukemia
Source: Nat Commun. 2019 Oct 25;10:4869. doi: 10.1038/s41467-019-12555-1 (PMC6814808; doi:10.1038/s41467-019-12555-1)
Supplement: Supplementary file 7 — Reporting Summary [file 41467_2019_12555_MOESM7_ESM.pdf]

nature research

Corresponding author(s): Toshio Kitamura
Susumu Goyama

Last updated by author(s): NCOMMS-19-03639B

Reporting Summary

Nature Research wishes to improve the reproducibility of the work that we publish. This form provides structure for consistency and transparency in reporting. For further information on Nature Research policies, see Authors & Referees and the Editorial Policy Checklist.

Statistics

For all statistical analyses, confirm that the following items are present in the figure legend, table legend, main text, or Methods section.

n/a	Confirmed	
☐	☒	The exact sample size (*n*) for each experimental group/condition, given as a discrete number and unit of measurement
☐	☒	A statement on whether measurements were taken from distinct samples or whether the same sample was measured repeatedly
☐	☒	The statistical test(s) used AND whether they are one- or two-sided<br>*Only common tests should be described solely by name; describe more complex techniques in the Methods section.*
☒	☐	A description of all covariates tested
☒	☐	A description of any assumptions or corrections, such as tests of normality and adjustment for multiple comparisons
☐	☒	A full description of the statistical parameters including central tendency (e.g. means) or other basic estimates (e.g. regression coefficient) AND variation (e.g. standard deviation) or associated estimates of uncertainty (e.g. confidence intervals)
☐	☒	For null hypothesis testing, the test statistic (e.g. *F*, *t*, *r*) with confidence intervals, effect sizes, degrees of freedom and *P* value noted<br>*Give P values as exact values whenever suitable.*
☒	☐	For Bayesian analysis, information on the choice of priors and Markov chain Monte Carlo settings
☒	☐	For hierarchical and complex designs, identification of the appropriate level for tests and full reporting of outcomes
☒	☐	Estimates of effect sizes (e.g. Cohen's *d*, Pearson's *r*), indicating how they were calculated

Our web collection on statistics for biologists contains articles on many of the points above.

Software and code

Policy information about availability of computer code

Data collection	Not applicable.
Data analysis	FlowJo 10.3.<br>GSEA MSigDB (http://software.broadinstitute.org/gsea/msigdb)<br>SPRING (https://kleintools.hms.harvard.edu/tools/spring.html)<br>GraphPad Prism 7<br>FastQC<br>Bowtie software (http://bowtie-bio.sourceforge.net/index.shtml/)<br>SAM tools (http://samtools.sourceforge.net/)<br>TopHat software (http://tophat.cbcb.umd.edu/)<br>Cufflinks software (http://cufflinks.cbcb.umd.edu/)

For manuscripts utilizing custom algorithms or software that are central to the research but not yet described in published literature, software must be made available to editors/reviewers. We strongly encourage code deposition in a community repository (e.g. GitHub). See the Nature Research guidelines for submitting code & software for further information.

Data

Policy information about availability of data

All manuscripts must include a data availability statement. This statement should provide the following information, where applicable:

- Accession codes, unique identifiers, or web links for publicly available datasets
- A list of figures that have associated raw data
- A description of any restrictions on data availability

The RNA-sequencing data have been deposited in the NCBI gene expression omnibus under the accession code GSE135008[https://www.ncbi.nlm.nih.gov/geo/query/acc.cgi?acc=GSE135008]. The Mass-cytometry data referenced during the study are available in Mendeley Data (http://dx.doi.org/10.17632/fsrwv9hpt8.1).

All the other data supporting the findings of this study are available within the article and its supplementary information files and from the corresponding author upon reasonable request. A reporting summary for this article is available as a Supplementary Information file.

Field-specific reporting

Please select the one below that is the best fit for your research. If you are not sure, read the appropriate sections before making your selection.

☒ Life sciences ☐ Behavioural & social sciences ☐ Ecological, evolutionary & environmental sciences

For a reference copy of the document with all sections, see nature.com/documents/nr-reporting-summary-flat.pdf

Life sciences study design

All studies must disclose on these points even when the disclosure is negative.

Sample size	No statistical methods were used to predetermine sample sizes. We ensured that they were similar to those generally employed in the field.
Data exclusions	For SPRING analysis, the data (FSC files) were downsampled from 100,000 into 3,000 cell events due to software capacity limit.
Replication	All experiments were repeated at least twice.
Randomization	All mice were randomized before tumor inoculation.
Blinding	Blinding was not performed in this study.

Reporting for specific materials, systems and methods

We require information from authors about some types of materials, experimental systems and methods used in many studies. Here, indicate whether each material, system or method listed is relevant to your study. If you are not sure if a list item applies to your research, read the appropriate section before selecting a response.

Materials & experimental systems

n/a	Involved in the study	
☐	☒	Antibodies
☐	☒	Eukaryotic cell lines
☒	☐	Palaeontology
☐	☒	Animals and other organisms
☐	☒	Human research participants
☒	☐	Clinical data

Methods

n/a	Involved in the study	
☒	☐	ChIP-seq
☐	☒	Flow cytometry
☒	☐	MRI-based neuroimaging

Antibodies

Antibodies used	All antibodies used in the manuscript were purchased from commercial sources (details for each antibody are provided in the methods section).
Validation	Antibodies were validated individually using positive and negative controls.

Eukaryotic cell lines

Policy information about cell lines

Cell line source(s)	293T cell (ATCC), Plat-E cell [We generated it. (Gene Therapy 7(12):1063-6, 2000)]
Authentication	We used low-passage cells.
Mycoplasma contamination	We performe regular testing for mycoplasma contamination.
Commonly misidentified lines (See ICLAC register)	No commonly misidentified lines were used in this study.

Animals and other organisms

Policy information about studies involving animals; ARRIVE guidelines recommended for reporting animal research

Laboratory animals	C57BL/6J mice<br>p53–/– mice<br>Hif1a flox/flox mice<br>Rosa26-Cre-ERT2 mice<br>Rosa26-LSLCas9 knockin mice<br>C57BL/6-Tg(CAG-EGFP) mice<br>NSG mice<br>NSGS mice
Wild animals	Not applicable.
Field-collected samples	Not applicable.
Ethics oversight	All experiments in this study were performed according to an institutional review board-approved protocol, in accordance with the Declaration of Helsinki, and with an approved animal study IACUC protocol at CCHMC or the Animal Care Committee of the Institute of Medical Science at the University of Tokyo.

Note that full information on the approval of the study protocol must also be provided in the manuscript.

Human research participants

Policy information about studies involving human research participants

Population characteristics	All information for human material is provided in Supplementary Table 1.
Recruitment	Residual diagnostic specimens from AML patients who acquired informed consent properly at Cincinnati Children's Hospital Medical Center were used.
Ethics oversight	Informed consent was properly acquired at Cincinnati Children's Hospital Medical Center.

Note that full information on the approval of the study protocol must also be provided in the manuscript.

Flow Cytometry

Plots

Confirm that:

- [x] The axis labels state the marker and fluorochrome used (e.g. CD4-FITC).
- [x] The axis scales are clearly visible. Include numbers along axes only for bottom left plot of group (a 'group' is an analysis of identical markers).
- [x] All plots are contour plots with outliers or pseudocolor plots.
- [x] A numerical value for number of cells or percentage (with statistics) is provided.

Methodology

Sample preparation	Bone marrow cells were obtained by either crushing or flushing femurs and tibias in PBS containing 2% FBS40. Red blood cells were removed using RBC lysis buffer. Cells were then stained by fluoro-conjugated antibodies for 30min at 4ºC. After staining, cells were washed with cold PBS for several times, and were resuspended with PBS containing 2% FBS.
Instrument	Cells were analysed on a FACS Calibur or a FACS Verse, and were sorted with a FACSAria (BD Biosciences, San Jose, CA, USA).
Software	FlowJo 10.3.
Cell population abundance	The number of capture was 10,000 counts unless there are special circumstances.
Gating strategy	Sequential gating/sorting strategies are provided in Supplementary Figure 13.

- [x] Tick this box to confirm that a figure exemplifying the gating strategy is provided in the Supplementary Information.
